# DFT Benchmarking for [Fe^II^]‑(Alkyl/Alkylidene/Acetylide) Complexes

**DOI:** 10.1021/acsomega.5c00517

**Published:** 2025-12-08

**Authors:** Leonardo de S. da Silva, Franciscarlos S. Soares, Alexandre A. de Souza, Égil Sá

**Affiliations:** † Laboratório de Química Teórica, Universidade Federal do Piauí, Teresina, PI 64049-505, Brazil; ‡ Departamento de Química, Universidade Federal do Piauí, Teresina, PI 64049-505, Brazil

## Abstract

Given the growing significance of earth-abundant organometallic catalysis, especially with iron, this study provides a comprehensive evaluation of the most effective exchange–correlation functionals (XCFs) for accurately reproducing the experimental geometry, ground spin-state, and reactivity of organometallic Fe^II^ complexes bearing alkyl, alkylidene, or acetylide ligands. We optimized these complexes in various spin-states using twelve different XCFs and compared the results with experimental data. Subsequently, we analyzed the optimized geometries of the most stable spin-states against the experimental structures. Finally, we evaluated the ability of the XCFs to replicate the experimental stereoselectivity of the cyclopropanation reaction. Our results indicate that among the functionals evalueted, B3LYP* and r^2^-SCAN are the XCFs that most effectively reproduce the experimental observations considered for the systems evaluated in this work.

## Graphical abstract

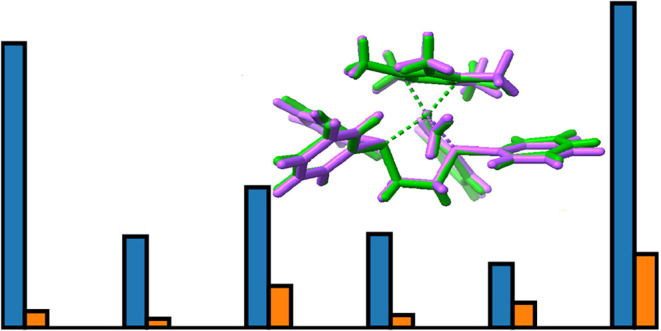

## Introduction

Organometallic chemistry serves as a remarkable toolbox in the field of catalysis. Over the past decade, this discipline has undergone a significant transformation, shifting focus from noble and precious metal systems to those based on earth-abundant metals.^1,2^ Among these, iron complexes have been explored as potential catalysts for a wide range of applications.^3–6^ One potential interesting application of iron-based systems is olefin metathesis,^7^ where iron-carbenes are often proposed as promising catalysts. Beyond this, iron-carbenes have demonstrated catalytic potential in stoichiometric cyclopropanation^8^ and have been employed in engineered biocatalysts for a variety of carbene transfer reactions,^9–12^ such as hydroaminations, hydroborations, hydrosilylations, S–H insertions, C–H functionalization, aldehyde olefinations, sigmatropic rearrangements, cyclopropenations, and bicyclobutanations.

The versatility of [Fe]-alkylidene underscores the importance of further investigating these systems and highlights the necessity of employing appropriate theoretical frameworks to study them. One distinguishing feature of 3d metals, such as iron, is their tendency to exhibit spin-states other than the low-spin-state. This phenomenon arises because their d-orbitals are more compact compared with those of 4d and 5d metals, which hinders electron pairing. Consequently, while singlet states are often the ground state for 4d and 5d metals, this is less commonly observed in 3d metals.^13^

Amid this effort, computational contributions are central to the development of the field. In this sense, it is relevant to establish which would be the proper level of theory to describe these systems, especially focusing on which exchange–correlation DFT functional is more suitable. There are some previous benchmark contributions for iron complexes. One of the first works in this regard, done by Swart and co-workers, showed that for penta- and hexacoordinated complexes, OPBE is the most suitable exchange–correlation DFT functional (XCF) able to reproduce the experimental spin-state and geometry.^14^ Other contributions, focusing on the spin crossover of iron­(II) and iron­(III), have shown that B3LYP is the best XCF to reproduce the phenomena,^15,16^ others,^17,18^ again point out OPBE, along with B3LYP, while efforts^19^ embracing another 3d metals show TPPSh as the proper functional. In another analysis, 31 functionals were considered over 5 hexacoordinated complexes; the authors found that, again, OPBE appears as efficient along with B2PLYP.^20^ A modification of the amount of exact exchange (15% instead of 20%) for the B3LYP, called B3LYP*, was proven to be useful to study excited states in two iron systems^21^ and spin crossover.^22^ It was shown that the S12g functional was suitable to describe oxoiron systems.^23^ It was also claimed that among a few XCF, ω-B97XD performed best to describe iron porphyrin carbenes.^24^ Therefore, as for these works, throughout different properties and systems, there is no agreement on which is the proper XCF to treat the systems we are focusing on in this work.

Recently, some benchmarking efforts^25–28^ have highlighted the relevance of benchmarking works that take experimental quantities as reference, instead of high-level calculations, such as CCSD­(T), which is also the approach chosen for this work. In this way, this contribution aims to provide a straightforward benchmarking against experimental parameters of the most common XCFs to reproduce spin-states, geometries, and kinetics of organometallic Fe^II^ complexes, bearing alkyl, alkylidene and acetylide as pivotal ligands.

## Approach and Methodology

Herein, we compare the DFT calculated results to the experimental results, regarding the structure and spin-states of 12 complexes of Fe^II^ with one alkyl, alkylidene, or acetylide as pivotal ligand, in the sense that this ligand is responsible for the reactivity exhibited by the given complex. We have gathered, to the best of our knowledge, the experimentally reported complexes within these conditions, with reported X-ray structure and ground spin-state. The complexes considered in this work are listed in Scheme 1.

**Scheme 1:**
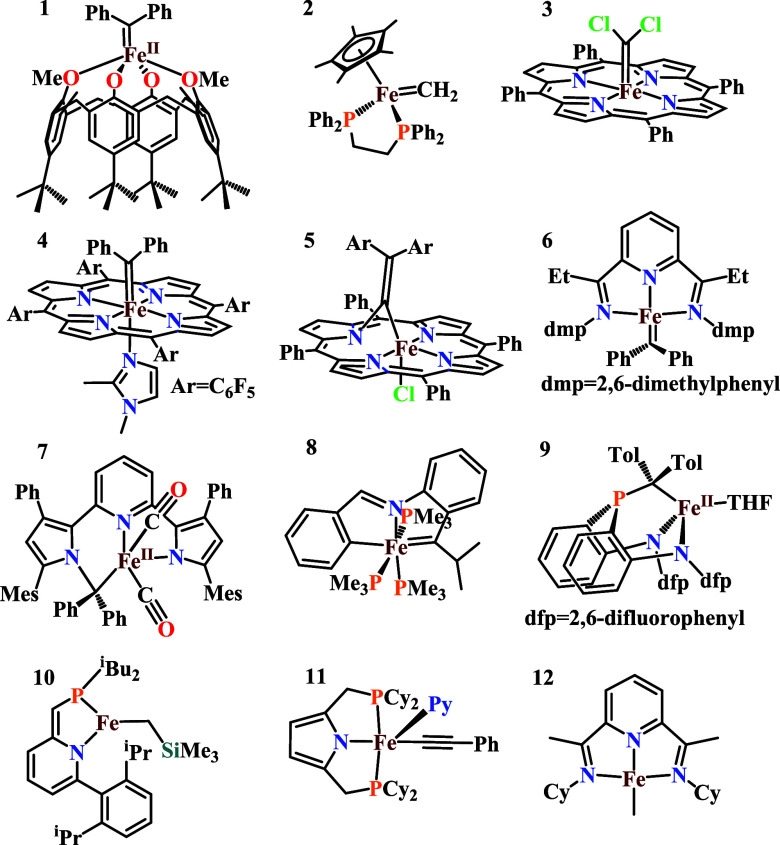
[Fe^II^] Organometallic Complexes Analyzed in This Work, Bearing Alkyl, Alkylidene, and Acetylide as Pivotal Ligands^a^ ^40^

a References Are **1**,^29^ **2**,^30^ **3**,^31^ **4**,^32^ **5**,^33^ **6**,^34^ **7**,^35^ **8**,^36^ **9**,^37^ **10**,^38^ **11**,^39^ and **12**.^40^.

For each XCF, the geometries were optimized in the gas phase without any geometrical constraints, including dispersion corrections. The optimizations were carried out in the singlet, triplet, and quintet spin-states. Main group elements were represented with the valence double-ζ plus polarization basis set 6–31G­(d,p),^41,42^ and iron was represented by the valence triple-ζ Wachters-Hay’s plus polarization basis set enlarged with diffuse functions 6–311+G­(d,p).^41,43–46^ Calculations for open-shell systems were carried out considering the spin-unrestricted formalism. Frequency calculations were performed to determine the nature of the stationary points. These frequency calculations were also used to obtain thermodynamic energies at 298.15 K and 1 atm. So far, we call this calc1. To improve accuracy, the final energetics were obtained by single-point calculations employing the 6–311++G­(d,p)^41,43–46^ basis set on the optimized geometries from the previous step, including dispersion corrections and solvent effects by means of SMD solvent continuous calculations, based on the solvents used in the experiments (see Table S1 in the Supporting Information). We call this calc2. All of the energies reported across the text are Gibbs Free Energies, which are defined as the summation of the single-point energies (calc2) plus the Gibbs’ thermal corrections and zero-point energy corrections obtained in the optimizations of the previous step (calc1).

We have chosen the most popular XCF functionals in the rungs of the Jacob ladder.^47^ Some of them were taken because they have already been reported as suitable to treat iron systems: BP86 ^48,49^ and OPBE^50^ for the rung of the generalized gradient approximation (GGA); M06-L,^51^ since it was designed to organometallic systems, TPSS^52^ and the recent r^2^-SCAN^53^ in the rung of meta-GGA; B3LYP,^48,54,55^ B3LYP*,^56^ and PBE0 ^57^ belonging to hybrid-GGA; TPSSh,^58^ M05,^59^ M06 ^60^ representing hybrid meta-GGA; last ω-B97XD^61^ from the range-separated hybrid-GGA. Grimme’s GD3 dispersion corrections were taken into account for all calculations, for both optimization and single-point, as implemented in Gaussian 09-Revision D1,^62^ where the parameters are implemented from ref 63 for XCFs BP86, B3LYP, TPSS, PBE0, and the parameters to M06, M06-L and M05 are from ref 64. The parameters were manually inserted for XCFs TPSSh, OPBE, also from ref 64, and for ω-B97XD from ref 65. Given the inexpensiveness and spread usage, we also analyzed the semiempirical method GFN2-xTB.^66^ All calculations were performed with the Gaussian 09 package,^62^ except for r^2^-SCAN and GFN2-xTB, which were done with Orca.^67,68^ For open-shell calculations with ⟨*S* ^2^⟩ value larger than 10%, we did spin-contamination corrections,^69^ as shown in Section S2, in the Supporting Information.

## Results and Discussion

The results of this study are systematically presented and thoroughly discussed across three distinct aspects: initially, an exploration of the reproducibility of spin-states, followed by an in-depth analysis of the structural and geometric characteristics of the complexes, and finally, a comprehensive evaluation of the kinetic properties.

### Spin Ground State Reproducibility

For systems containing an odd number of electrons (**5** and **12**), we systematically explored the doublet, quartet, and sextet spin-states to identify the most stable configuration. For systems with an even number of electrons, we considered singlet, triplet, and quintet spin-states. The singlet state to which we are referring is the spin-restricted solution. We also performed calculations for the open-shell singlet solution, however, in no case was this solution the ground state. Therefore, we decided not to include these energies in the discussion. The resulting energies from these calculations, which provide a comprehensive overview of spin-state stability across all systems, are summarized in Table 1.

**Table 1:** Calculated Spin-State Energies (kcal·mol^–1^) with Exchange–Correlation Functionals for Different Fe^II^ Complexes^a^

complex		1	2	3	4	5	6	7	8	9	10	11	12
exp. spin (S)^b^		2	0	0	0	3/2	1	0	0	2	2	1	3/2
exp. method^c^		μ_B_ ^d^	EPR^e^	Möss^f^	Möss^f^	EPR^e^	Möss^f^	Möss^f^	Möss^f^	Möss^f^	Möss^f^	μ_B_ ^d^	Möss^f^
BP86	LS	0.00^g^	0.00	0.00	0.00	0.00	0.00	0.00	0.00	0.00	0.00	0.00	***0.00*** ^h^
	IS	–17.27	22.45	20.39	24.25	–3.77	–1.24	14.01	14.10	–12.63	–28.35	–7.35	5.60
	HS	–26.90	48.27	31.24	36.11	15.61	7.66	27.01	33.78	–19.30	–26.02	17.23	21.35
OPBE	LS	0.00	0.00	0.00	0.00	0.00	0.00	0.00	0.00	0.00	0.00	0.00	***0.00***
	IS	–26.45	23.93	18.32	19.49	–13.06	–6.51	14.22	16.77	–16.61	***–32.87***	–7.53	0.14
	HS	–42.48	49.16	17.18	23.68	–1.07	–0.60	26.96	34.96	–27.20	–22.77	13.33	11.53
TPSS	LS	0.00	0.00	0.00	0.00	0.00	0.00	0.00	0.00	0.00	0.00	0.00	***0.00***
	IS	–16.55	19.31	15.73	15.63	–3.65	–3.97	14.93	15.23	–13.81	***–31.71***	–8.81	2.31
	HS	–28.66	42.29	26.01	33.78	14.04	4.52	23.93	31.40	–21.99	–31.54	11.04	17.01
M06-L	LS	0.00	0.00	0.00	0.00	0.00	0.00	0.00	0.00	0.00	0.00	0.00	0.00
	IS	–27.04	14.88	12.29	19.19	–4.86	–12.66	9.62	10.75	–14.77	–26.37	–15.45	–21.29
	HS	–56.29	23.35	0.40	2.07	–3.70	–18.79	9.37	13.36	–33.03	–43.82	–12.86	–6.34
r^2^-SCAN	LS	0.00	0.00	0.00	0.00	0.00	0.00	0.00	0.00	0.00	0.00	0.00	0.00
	IS	–28.66	13.68	43.57	21.43	–12.15	–0.78	7.07	10.58	–12.33	–24.04	–13.69	–7.75
	HS	–42.34	35.37	58.41	16.79	9.00	1.84	20.62	25.02	–31.46	–34.02	20.42	4.50
B3LYP	LS	0.00	0.00	0.00	0.00	0.00	0.00	0.00	0.00	0.00	0.00	0.00	0.00
	IS	–24.73	10.56	6.51	15.83	–12.29	–9.95	3.54	8.50	–16.52	–39.28	–15.06	–33.21
	HS	–45.42	18.40	3.13	5.90	–4.35	–9.02	6.00	8.40	–28.81	–53.71	–8.72	–15.35
B3LYP*	LS	0.00	0.00	0.00	0.00	0.00	0.00	0.00	0.00	0.00	0.00	0.00	0.00
	IS	–22.87	13.52	20.08	16.81	–10.27	–18.26	6.67	1.01	–16.33	–36.61	–12.96	–18.74
	HS	–40.82	24.70	9.67	11.37	1.79	–5.86	2.96	13.28	–33.47	–48.36	–2.32	–3.34
PBE0	LS	0.00	0.00	0.00	0.00	0.00	0.00	0.00	0.00	0.00	0.00	0.00	0.00
	IS	–32.01	7.59	15.18	23.27	–19.36	–12.47	2.13	6.22	–20.25	–44.65	–17.41	–16.78
	HS	–55.37	15.23	***–5.53***	2.47	–15.58	***–14.33***	2.65	6.56	–45.36	–66.05	–13.93	***–26.09***
TPSSh	LS	0.00	0.00	0.00	0.00	0.00	0.00	0.00	0.00	0.00	0.00	0.00	0.00
	IS	–21.37	14.45	19.77	13.45	–11.65	–12.24	11.43	11.01	–17.57	–37.83	–11.69	–7.59
	HS	–38.17	31.66	13.67	22.69	0.85	–3.71	13.93	21.90	–24.08	–44.15	2.65	1.65
M05	LS	0.00	0.00	0.00	0.00	0.00	0.00	0.00	0.00	0.00	0.00	0.00	0.00
	IS	–42.77	9.80	–0.28	–2.40	–23.20	–19.99	–4.25	4.64	–19.96	–39.17	–25.90	–30.30
	HS	–74.71	9.57	***–21.90***	***–26.08***	***–25.75***	***–36.27***	***–7.87***	***–8.30***	–55.92	–62.52	***–28.18***	–23.38
M06	LS	0.00	0.00	0.00	0.00	0.00	0.00	0.00	0.00	0.00	0.00	0.00	0.00
	IS	–30.19	8.65	21.07	13.23	–19.15	–5.76	–0.89	8.88	–21.69	–38.66	–19.40	–25.57
	HS	–64.90	9.54	***–16.26***	***–12.42***	–17.40	13.34	***–1.25***	3.28	–50.07	–61.61	–20.29	–17.42
ω-B97XD	LS	0.00	0.00	0.00	0.00	0.00	0.00	0.00	0.00	0.00	0.00	0.00	0.00
	IS	6.52	9.46	21.89	27.53	–4.66	1.19	2.07	5.91	–17.52	–32.42	–15.79	–36.33
	HS	–43.16	20.50	26.66	23.94	18.02	***–18.01***	52.62	8.42	–39.84	–53.34	–11.21	–29.18

a LS stands for singlet, IS for triplet, and HS for quintet. In the case of the complexes with an odd number of electrons (**5** and **12**), LS, IS, and HS stand for the doublet, quartet, and sextet, respectively.

b Ground spin-state experimentally determined.

c Method used to determine the experimental ground spin-state.

d Magnetic susceptibility, measured by the Evans method.

e Electronic Paramagnetic Resonance spectroscopy.

f Mössbauer Spectroscopy.

g The energy of the singlet spin-state is taken as the reference for all systems with an even number of electrons, while the doublet is taken as the reference in the case of odd number of electrons.

h The system where a given XCF does not reproduce the experimental spin-state is in bold-italic.

We consider an experimental spin-state to be successfully reproduced by the calculations of a given XCF when the most stable geometry, among the calculated spin-states, coincides with the experimentally observed spin-state. The results reveal that B3LYP, B3LYP*, M06-L, TPSSh, and r^2^-SCAN successfully reproduced all of the experimental spin-states. This is partially in agreement with previous studies where TPPSh appear as proper functional to model spin-crossing.^19^ As expected, a small amount of exact exchange in B3LYP* promotes higher energies, regarding B3LYP, for intermediate and high spin-states: a paradigmatic example is complex **12** quartet that for B3LYP has a relative energy of −33.21 kcal·mol^–1^ and for B3LYP* is −18.74 kcal·mol^–1^. Eight XCFs failed to match the experimental spin-state in two systems: BP86 (complexes **8** and **12**), OPBE (complexes **10** and **12**), TPSS (complexes **8** and **12**), and ω-B97XD (complexes **4** and **6**). PBE0 failed in three cases (complexes **3**, **6**, and **12**), while M06 failed in four instances (complexes **3**, **4**, **7**, and **11**). As expected, M05 performed badly, failing to reproduce the ground spin-state in seven out of 12 systems (complexes **3**, **4**, **5**, **6**, **7**, **8**, and **11**). In summary, B3LYP, B3LYP*, M06-L, TPSSh, and r^2^-SCAN are the most reliable XCFs for determining the ground spin-state among those evaluated in this study. Please note that these calculations do not consider the relativistic effects of the iron atoms. Including them by means of effective core potential SDD^70^ (see the Supporting Information Table S4) leads to a larger number of errors in reproducing the ground spin-state.

The exact exchange percentage refers to the fraction of Hartree–Fock exchange included in a given density functional. Among the functionals used in this study, BP86, OPBE, TPSS, M06-L, and r^2^SCAN contain 0% exact exchange. B3LYP and PBE0 incorporate 20 and 25% exact exchange, respectively, while B3LYP* is a modified version with 15%. TPSSh is a hybrid meta-GGA with a 10% exact exchange. M06 and M05 have higher exact exchange contents of 27 and 28%, respectively. Lastly, ωB97XD is a range-separated hybrid functional that includes approximately 22% short-range exact exchange.

Our results reveal that the percentage of exact exchange (HF) in a functional could not be the sole factor determining its accuracy in predicting spin-state energetics. While M06-L and r^2^SCAN (0% exact exchange) performed perfectly, demonstrating that advanced meta-GGAs can achieve high accuracy without exact exchange, other pure GGAs/meta-GGAs such as BP86, OPBE, and TPSS failed in some cases. XCFs with moderate exact exchange (10–20%)—TPSSh, B3LYP*, and B3LYP—were consistently successful, suggesting an optimal balance. In contrast, higher exact exchange (>25%) in M05, M06, and PBE0 led to poorer performance. Thus, both the functional type (GGA/hybrid-GGA/meta-GGA/range-separated) and the amount of exact exchange play critical roles. Our data seems to indicate that the best results, in determining the ground spin-state, come from well-balanced hybrids (10–20%) or sophisticated meta-GGAs (0%).

### Geometry Reproducibility

Another part of our approach is to analyze the ability of the XCF to reproduce the experimental geometry of the complexes using the X-ray structure as a reference. To do this, we selected the most stable optimized spin-state structure for each complex. We then analyzed the RMSD for the positions of the atoms for each XCF, as shown in Figure 1. The RMSD was calculated according to eq 1, where *N* is the number of atoms in each system and δ*_i_* represents the difference between a Cartesian position of a given atom calculated for a given XCF optimization structure and the corresponding Cartesian position in the experimental X-ray structure. Given the critical role of the first coordination sphere in organometallic chemistry, we also assessed the RMSD for these regions in each complex, which is depicted in Figure 1, according to the definition of the first coordination sphere in Figure S1, in the Supporting Information. Because the positions of the hydrogens in the resolved X-ray crystal structures are imprecise, we decided to consider only the heavy atoms in our geometrical analysis, that is, all of the atoms that are not hydrogens.^71,72^ Indeed, Figure S2 shows that if the hydrogens are not considered, the RMSDs are systematically smaller than those where hydrogens are included. The RMSD calculation of the whole system with and without hydrogen was performed as implemented in Chemcraft, and the RMSD of the first coordination sphere was done with a script.

1 $$\mathrm{RMSD}=\sqrt{\frac{1}{N}\sum_{i=1}^{N}\delta_{i}^{2}}$$

**Figure 1:**
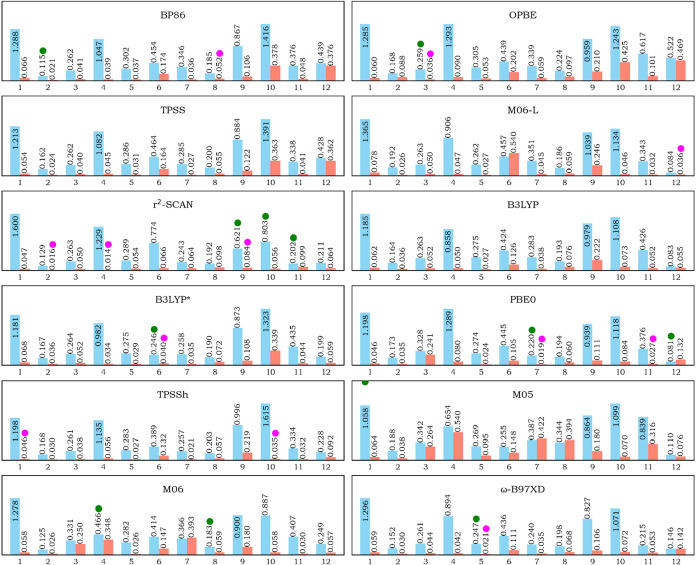
RMSD (Å) of the most stable spin-state of each complex for each XCF, for all of the atoms, hydrogens atoms excluded (cyan square), and for the first coordination sphere (orange square), indicating the XCF with the smallest RMSD when the entire system is considered (green circle), and when only the first coordination sphere (pink circle).

For the RMSD of the entire systems, r^2^-SCAN performed the best in reproducing the largest number of systems, specifically complexes **9**, **10**, and **11**. Three methods excelled in reproducing two complexes each: PBE0 for complexes **7** and **12** and M06 for complexes **4** and **8**. Other methods, BP86, OPBE, B3LYP*, M05, and ω-B97XD, performed best for one system each: complexes **2**, **3**, **6**, **1**, and **5**, respectively. Neither TPSS, B3LYP, nor TPPSh was the top-performing method for any system. Considering the inner coordination sphere, again, r^2^-SCAN is the XCF that performs best for most systems, which are **2**, **4**, and **9**. TPPSh is the best XCF for systems **1** and **10**, as well as PBE0, for systems **7** and **11**. BP86, OPBE, M06-L, B3LYP*, and ω-B97XD show the smallest RMSD for only one system each, which are **8**, **3**, **12**, and **5**, respectively. Neither for the inner coordination sphere nor for the whole system have TPSS and B3LYP shown the best RMSD.

However, it might be better to consider not only the best XCF for each complex but also the best XCF across all complexes in order to assess their consistency, even if they do not yield the smallest RMSD for an individual complex. To this end, we summed the RMSD (∑RMSD) for each XCF, considering both the entire system and the inner coordination sphere, followed by the ranking, as shown in Figure 2. For the entire system, M06 exhibits the smallest ∑RMSD, followed by ω-B97XD and B3LYP. For the first coordination sphere, r^2^-SCAN gives the smallest ∑RMSD, followed by ω-B97XD and TPSSh.

**Figure 2:**
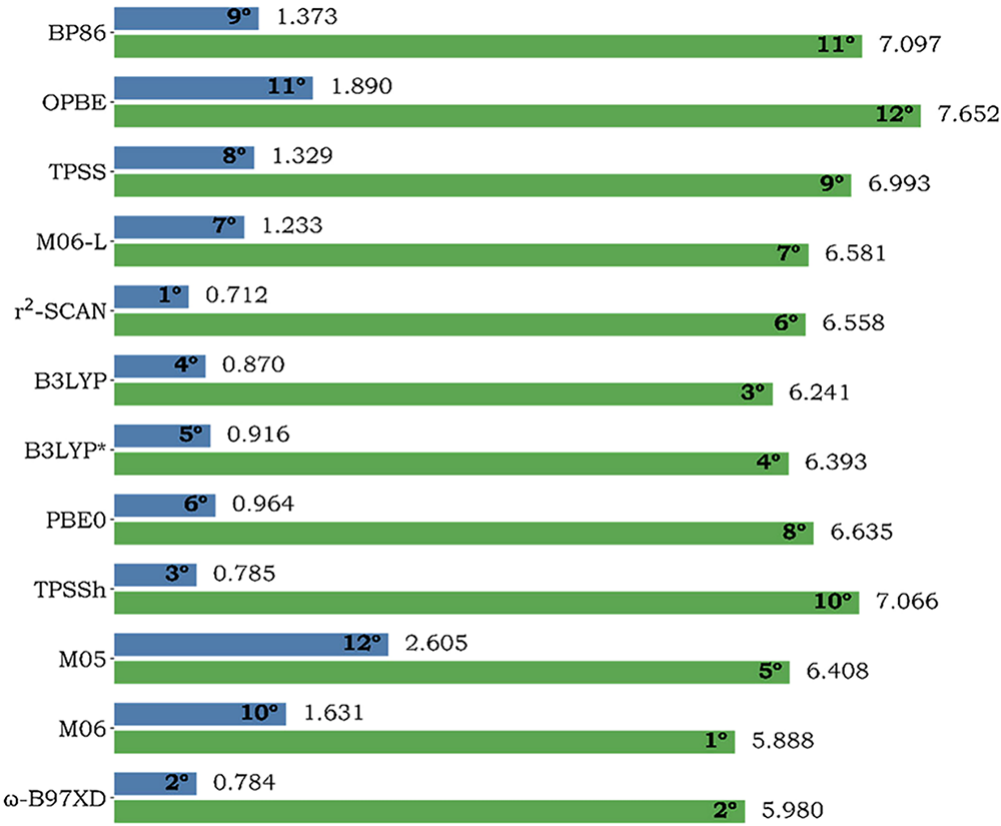
∑RMSD (Å) for each exchange–correlation functional, considering both the entire system (green) and the first coordination sphere only (blue), showcasing the RMSD values at the side of each bar along with the rank of each XCF.

Although a clear trend could not be assigned, based on the ability to reproduce experimental geometries, the data indicates that r^2^-SCAN is the most suitable XCF among those considered in this study, since it best reproduces the geometry of three systems when considering all of the atoms, and three systems when considering the first coordination sphere. r^2^-SCAN also showed consistency across the systems, as its ∑RMSD for the first coordination sphere (0.712 Å) is the smallest, and because the first coordination sphere is of more relevance for the reactivity and properties of organometallic systems, we take it as more important to determine the most suitable XCF. Figure S3 (in the Supporting Information) shows the superposition of the X-ray experimental structure and the optimized structure with r^2^-SCAN. On the other hand, M05 and OPBE appeared to be the least effective XCFs, considering the whole systems and the first coordination sphere, respectively.

### Kinetic Reactivity Reproducibility

In order to assess the capability of the XCF to reproduce the reactivity patterns of iron-carbenes, we performed a comparison with experimental stereoselectivity data. Specifically, we examined the reactivity of both piano-stool (**13**) and heme iron-carbenes (**14**, **15**, **16**), as defined in Scheme 2, with olefins in a stoichiometric cyclopropanation reaction, with a particular focus on *cis*/*trans* stereoselectivity. Scheme 2 shows the general reaction for cyclopropanation, generating *cis* and *trans* products. Substituents R and R″ depend on the carbene and the olefin. Experimentally, the heme iron-carbenes^73–75^ (**14**, **15**, **16**) predominantly generate the *trans*-cyclopropanes, while, rather unexpectedly, the piano-stool iron-carbenes^76^ (**13**) produce the less thermodynamically stable *cis*-cyclopropanes.

**Scheme 2:**
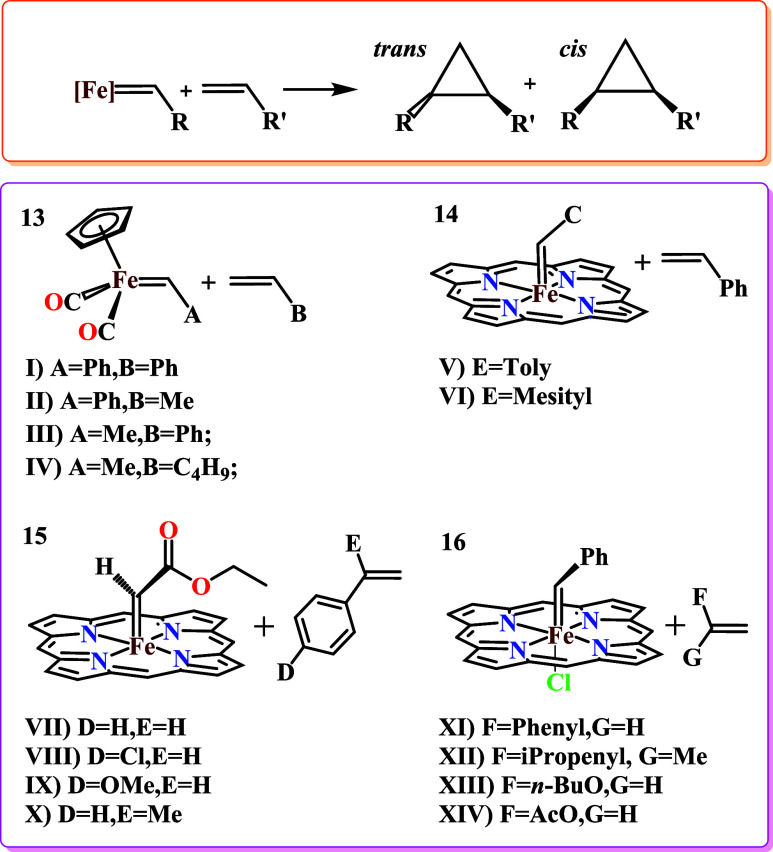
General Reaction of Cyclopropanation by Carbene Transfer from [Fe^II^]-Carbenes to Olefins, Resulting in *cis*/*trans* Products (top), along with the Reactions Considered in This Work to Analyze the Stereoselectivity Reproducibility (Bottom)

DFT (OPBE) calculations^77^ have demonstrated that these reactions are kinetically controlled, with the formation of the less stable *cis*-cyclopropanes, in the case of complex **13**, resulting from the presence of attractive noncovalent interactions. These interactions stabilize the *cis* transition state relative to the *trans* transition state in the rate-determining step of the reaction. The same study^77^ also revealed that the transition state associated with the rate-determining step occurs in the singlet state, which is the ground spin-state that we consider in the calculations for this part of the work. It is important to note that, to the best of our knowledge, there is no available experimental X-ray data or ground spin-state determination for complexes **13**, **14**, **15**, and **16**. Since the reaction is kinetically controlled, an expression derived from the Arrhenius equation, assuming the same pre-exponential factor,^78–81^ (eq 2) can be applied, where Δ*G*_*cis*_^*#*^ represents the Gibbs free energy associated with the calculated transition state for the mechanism leading to the *cis* isomer, while Δ*G*_*trans*_^*#*^ corresponds to the *trans* isomer. Figure 3 shows the general aspects of the transition states considered.

$$\frac{\mathrm{cis}}{\mathrm{trans}}=\frac{v_{\mathrm{cis}}}{v_{\mathrm{trans}}}={e^{-}}^{\left(\Delta G_{\mathrm{cis}}^{\#}-\Delta G_{\mathrm{trans}}^{\#}\right)/\mathrm{RT}}$$ 2

**Figure 3:**
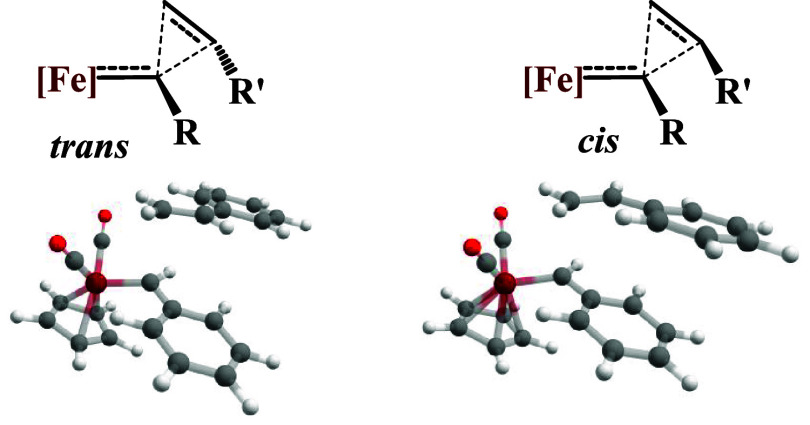
General form of the transition states involved in the reaction of cyclopropanation (top) and optimized geometries (r^2^-SCAN) for the reaction of catalyst **13** with styrene (bottom).

Experimental stereoselectivities were converted to Δ*G*_cis_^#^–Δ*G*_trans_^#^ values, herein referred to as ΔΔ*G*_exp_^#^, which served as the reference data. Figure 4 presents a heatmap comparing the calculated ΔΔ*G* ^#^ values from different exchange–correlation functionals (XCFs) against the experimental ΔΔ*G*_exp_^#^. The heatmap’s color scale is based on ranking the predicted values by their proximity to the experimental reference. For instance, in **system II**, the functional yielding the closest prediction (TPSSh) was assigned rank 1, followed by TPSS (rank 2), while the least accurate method (ω-B97XD) received rank 12. Extending this analysis across all complexes, r^2^-SCAN emerges as the best-performing XCF, achieving the top rank in three systems (**I**, **IX**, and **X**), second place in two systems (**VII** and **VIII**), and ranking last in only one system (**IV**). TPSSh was the top performer in the two systems (**II** and **III**), as were B3LYP (**IV** and **V**), B3LYP* (**VI** and **VIII**), and M06 (**XIII** and **XIV**). Across all systems examined, neither B3LYP nor B3LYP* ranked as the worst-performing XCF. In contrast, ω-B97XD appeared as the lowest-ranked functional in six systems, making it the worst overall performer. M06-L, BP86, and TPSS each had the lowest performance in two systems. OPBE was never either the best or the worst functional for any system in our evaluation. Overall, r^2^-SCAN, B3LYP, and B3LYP* demonstrated the best agreement with experimental stereoselectivities, while M05, M06, and especially ω-B97XD consistently appeared to be among the worst-performing methods.

**Figure 4:**
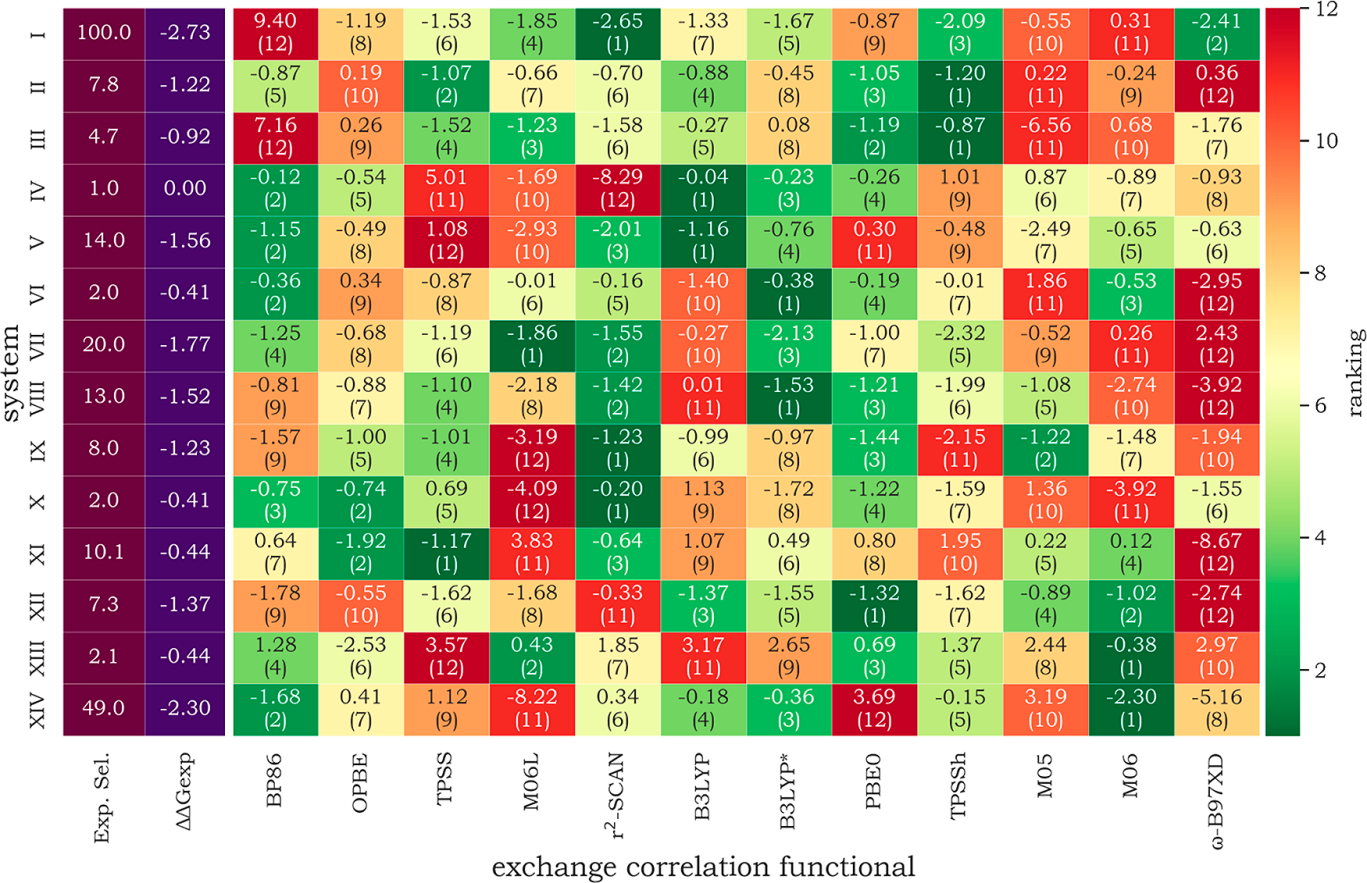
Calculated Δ*G*_*cis*_^*#*^–Δ*G*_*trans*_^*#*^ associated to the transitions states of the pathways that leads to *cis* and *trans* products. The values are energies in kcal·mol^–1^. The numbers in parentheses represent the performance ranking of each XCF, indicating how closely its predicted values match experimental results. The heatmap colors are based on this classification. The stereoselectivity experimentally observed is *cis/trans* for systems I–IV, VI, and XIV and *trans/cis* for systems V, VII–X.

To assess the consistency of each XCF through the whole set of systems, we calculated the mean absolute error (MAE) of ΔΔ*G* ^#^ predictions against experimental values, as summarized in Table 2. The baseline MAE (14p) includes all 14 data points for comprehensive evaluation. To examine outliers’ influence, we systematically excluded potentially off-range data points: 13p removes the single most divergent outlier that might otherwise obscure true performance, while 10p eliminates the four most extreme outliers for more robust error estimation. Additionally, 13No1 specifically excludes **system I**’s data point due to experimental indefiniteness, since the reported *cis*/*trans* stereoselectivity (>100)^76^ suggests potential measurement limitations that could distort the error analysis. It is important to note that the value of 100 (converted to ΔΔ*G* ^‡^ = −2.73 kcal·mol^–1^ in eq 2) is actually >100, and thus ΔΔ*G* ^‡^ < −2.73 kcal·mol^–1^. This tiered approach, perhaps, allows us to distinguish between genuine functional performance and artifacts and exceptional cases. See section S4 for additional data. MAE was calculated by eq 3, where *n* is the number of data points, ΔΔ*G*_*i XCF*_^#^ is the calculated Δ*G*_*cis*_^*#*^–Δ*G*_*trans*_^*#*^ for a given XCF, and ΔΔ*G*_*i* exp_^#^ is the experimental Δ*G*_*cis*_^*#*^–Δ*G*_*trans*_^*#*^.

3 $$\mathrm{MAE}=\frac{1}{n}\sum_{i=1}^{n}\left|\Delta \Delta G_{\mathrm{iXCF}}^{\#}-\Delta \Delta G_{i\;\exp}^{\#}\right|$$

**Table 2:** Mean Absolute Error (MAE) of Calculated ΔΔ*G*_XCF_^#^ Relative to Experimental ΔΔ*G*_exp_^#^ under Different Outlier Exclusion Criteria: Values Are in kcal·mol^–1^

**XCF**	**14p** ^a^	**13p** ^b^	**10p** ^c^	**13noI** ^d^
BP86	2.00	1.22	0.41	1.22
OPBE	1.05	0.92	0.70	1.02
TPSS	1.46	1.19	0.54	1.10
M06-L	1.72	1.40	0.74	1.33
r^2^SCAN	1.21	0.67	0.29	0.66
B3LYP	1.21	1.03	0.73	0.92
B3LYP*	0.94	0.77	0.49	0.93
PBE0	1.16	0.78	0.43	1.10
TPSSh	1.14	0.97	0.67	1.18
M05	1.93	1.65	1.08	1.91
M06	1.16	0.98	0.61	1.02
ωB97XD	2.19	1.80	1.30	2.34

a All 14 data points are included.

b Single most divergent outlier is excluded.

c Four most extreme outliers are excluded.

d System I data point is excluded due to experimental uncertainty.

Our MAE analysis agrees with our previous assessment, revealing consistent performance patterns across different data treatments, with B3LYP* and r^2^-SCAN emerging as the most reliable exchange–correlation functionals. When all 14 data points are evaluated, B3LYP* shows the lowest MAE (0.94 kcal·mol^–1^), followed by OPBE (1.05 kcal·mol^–1^) and TPSSh (1.14 kcal·mol^–1^). The robustness of these functionals emerges when examining outlier effects: excluding just one divergent data point causes r^2^-SCAN to surpass B3LYP* (0.67 vs 0.77 kcal·mol^–1^, respectively), with PBE0 (0.78 kcal·mol^–1^) ranking third. This trend strengthens when removing four potential outliers, where r^2^-SCAN achieves an accuracy of 0.29 kcal·mol^–1^, followed by B3LYP* (0.49 kcal·mol^–1^) and TPSS (0.54 kcal·mol^–1^). The analysis of **system I**’s impact further confirms r^2^-SCAN’s consistency (MAE = 0.66 kcal·mol^–1^ after exclusion), while revealing comparable secondary performance between M06 and OPBE (both 1.02 kcal·mol^–1^).

The MAE analysis consistently identifies ω-B97XD as the worst-performing functional across all four data treatments (14p, 13p, 10p, and 13No1). M05 shows particularly poor performance as well, ranking as the second-worst functional in three out of four cases (13p: 1.65 kcal·mol^–1^; 13No1: 1.91 kcal·mol^–1^; 10p: 1.076 kcal·mol^–1^). The only exception occurs in the full 14-point data set (14p), where BP86 claims the position of second-worst performer with an MAE of 2.00 kcal·mol^–1^. This pattern suggests fundamental limitations in these XCFs’ ability to accurately predict ΔΔG^#^ values, regardless of outlier inclusion or exclusion criteria. In summary, B3LYP* and r^2^SCAN emerge as the most reliable exchange–correlation functionals for kinetic studies, demonstrating favorable accuracy in energy predictions. In contrast, ω-B97XD and M05 exhibit significantly poorer performance.

In a separate analysis (Section S5), GFN2-xTB demonstrates that it is unsuitable for modeling iron-carbene systems. A comparative evaluation reveals that GFN2-xTB underperforms all examined exchange–correlation functionals (XCFs) across two critical aspects: (1) geometric structure prediction; (2) spin-state energetic. Regarding the kinetic calculation, GFN2-xTB showed moderate to poor performance. The method shows particularly pronounced deficiencies in reproducing accurate geometries and kinetic parameters, with deviations significantly exceeding those observed in DFT approaches. Its speed/accuracy balance makes it useful for preliminary studies.

## Conclusions

Given the increasing importance of earth-abundant elements, particularly iron-based organometallic systems due to their unique chemistry, this work aimed to recommend the most suitable exchange–correlation functionals (XCFs) for treating such systems by benchmarking them against experimental data. The XCFs exhibited varying levels of accuracy in predicting spin-states, geometries, and reactivity for the iron-carbene complexes studied. In terms of spin-state reproduction, B3LYP, B3LYP*, TPSSh, and r^2^-SCAN were the most reliable, as they were the only functionals capable of reproducing the experimental spin-states for all systems. Also, a higher amount of exact exchange (>25%) seems to lead to poor performance in spin-state reproduction. For kinetic predictions, B3LYP* and r^2^-SCAN emerged as the most suitable XCFs. r^2^-SCAN was also the most suitable for reproducing the experimental geometries. On the other hand, among the evaluated functionals, M05 demonstrated particularly poor performance across all examined properties. Notably, it failed to correctly reproduce spin ground states in the majority of test systems and exhibited the largest deviations in the inner coordination sphere summations, while OPBE showed greater deviations when considering complete systems. The kinetic energy reproducibility proved most problematic for both ω-B97XD and M05, which consistently ranked as the least accurate functionals in this study.

## Supplementary Material





## Notes

A .xyz file with all of the structures reported in the main text (XYZ)
Details of the calculations, spin-contamination data and its corrections, effective core potentials, and data of GFN2-xTB (PDF)
