# Supplementary material for: DFT Benchmarking for [FeII]‑(Alkyl/Alkylidene/Acetylide) Complexes
Source: ACS Omega. 2025 Dec 8;10(50):61169–78. doi: 10.1021/acsomega.5c00517 (PMC12750231; doi:10.1021/acsomega.5c00517)
Supplement: Supplementary file 2 [file ao5c00517_si_002.pdf]

DFT benchmarking for [Fe^{II}]-(alkyl/alkylidene/acetylide) complexes

Leonardo de S. da Silva.[a] *Franciscarlos S. Soares.*[a] *Alexandre A. de Souza*[a.b] *and Égil Sá**[a.b]

[a] Laboratório de Química Teórica. Universidade Federal do Piauí. Teresina-PI. 64049-505. Brazil

[b] Departamento de Química. Universidade Federal do Piauí. Teresina-PI. 64049-505. Brazil

Section S1 – Specific details for the calculations……………………………………………… S2

Section S2 – Additional data for Spin-State analysis. ……………………………………………… S8

Section S3 – Additional data for RMSD analysis……………………………………………… S13

Section S4 – Additional data used to kinetical analysis…………………...………………………… S16

Section S5 – Performance of GFN2-xTB……………………………………..……………………… S22

Section S8 – Influence of the solvent in the spin-state energetic……………………………………. S24

Section S1 – Specific details for the calculations.

Table S1. Solvents used in SMD calculations, according to the experimental solvents used, according to references 29-40 and 73-76 of the main text.

System	Solvent	System	Solvent
**I**	Dichloromethane	**VIII**	Diethyl Ether
**II**	Dichloromethane	**IX**	Benzene
**III**	Dichloromethane	**X**	Benzene
**IV**	Dichloromethane	**XI**	Tetrahydrofuran
**V**	Dichloromethane	**XII**	Diethyl Ether
**VI**	Diethyl Ether	**XIII**	Dichloromethane
**VII**	Diethyl Ether	**XIV**	Tetrahydrofuran

Representative examples of inputs for optimization calculations, minima and transition states, and single points calculations for energy refinement.

1) Gaussian 09 inputs for optimization calculations - (calc1).

1.1) Singlet

#p opt freq B3LYP/gen pop=full empiricaldispersion=gd3 gfinput scf=xqc 5D

B3LYP-1-S

0 1
xyz coordinates

C H O 0
6-31G(d,p)
****
Fe 0
6-311+G(d,p)
****

1.2) Triplet

#p opt freq B3LYP/gen pop=full empiricaldispersion=gd3 gfinput scf=xqc 5D

B3LYP-1-T

0 3
xyz coordinates

C H O 0
6-31G(d,p)
****
Fe 0
6-311+G(d,p)
****

1.3) Quintuplet

#p opt freq B3LYP/gen pop=full empiricaldispersion=gd3 gfinput scf=xqc 5D

B3LYP-1-Q

0 5
xyz coordinates

C H O 0
6-31G(d,p)
****
Fe 0
6-311+G(d,p)
****

1.4) Transition state in the singlet state

#p opt=(ts,calcfc,noeigentest) freq M06/gen pop=full empiricaldispersion=gd3 gfinput scf=xqc 5D

Heme Tolueno Estireno-cis

0 5
xyz coordinates

C H O 0
6-31G(d,p)
****
Fe 0
6-311+G(d,p)
****

2) Gaussian 09 inputs for single-point calculations aiming at energy refinement - (calc2).

2.1) Singlet

#p B3LYP/6-311++G(d,p) scf=direct EmpiricalDispersion=GD3 scrf=(smd,solvent=DiChloroMethane) 5D

B3LYP-1-S

0 1
xyz coordinates

2.2) Triplet

#p B3LYP/6-311++G(d,p) scf=direct EmpiricalDispersion=GD3 scrf=(smd,solvent=DiChloroMethane) 5D

B3LYP-1-T

0 3
xyz coordinates

2.4) Quintuplet

#p B3LYP/6-311++G(d,p) scf=direct EmpiricalDispersion=GD3 scrf=(smd,solvent=DiChloroMethane) 5D

B3LYP-1-Q

0 5
xyz

3) Orca 5.0 inputs for optimization calculations - (calc1).

3.1)Singlet

```
! OPT FREQ R2SCAN D3 6-31G(d,p) AUTOAUX TightSCF DEFGRID3 KDIIS NormalPrint

%maxcore 2500

%pal nproc 128 end

%scf
   MaxIter 500
end

%geom
   MaxIter 500
end

%basis
   NewGTO Fe "6-311+G(d,p)" end
end

%output
   print[p_mos] true
   print[p_basis] 5
end

*xyz 0 1
xyz coordinates
*
```

3.2) Triplet

```
! OPT FREQ R2SCAN D3 6-31G(d,p) AUTOAUX TightSCF DEFGRID3 KDIIS NormalPrint

%maxcore 2500

%pal nproc 128 end

%scf
   MaxIter 500
end

%geom
   MaxIter 500
end

%basis
   NewGTO Fe "6-311+G(d,p)" end
end

%output
   print[p_mos] true
   print[p_basis] 5
end

*xyz 0 3
xyz coordinates
*
```

3.3) Quintuplet

```
! OPT FREQ R2SCAN D3 6-31G(d,p) AUTOAUX TightSCF DEFGRID3 KDIIS NormalPrint

%maxcore 2500

%pal nproc 128 end

%scf
   MaxIter 500
end

%geom
   MaxIter 500
end

%basis
   NewGTO Fe "6-311+G(d,p)" end
end

%output
   print[p_mos] true
   print[p_basis] 5
end

*xyz 0 5
```
xyz coordinates
```
*
```

3.4) Transition state in the singlet state

```
! FREQ R2SCAN 6-31G(d,p) AUTOAUX TightSCF DEFGRID3 KDIIS NormalPrint

%maxcore 2500

%pal nproc 128 end

%scf
   MaxIter 500
end

%geom
   MaxIter 500
end

%basis
   NewGTO Fe "6-311+G(d,p)" end
end

%output
   print[p_mos] true
   print[p_basis] 5
end

*xyz 0 5
```
xyz coordinates
```
*
```

For the calculations of the modification version of B3LYP with 15% of exact exchange (B3LYP*) the following keyword was added:

IOp(3/76=1000001500,3/77=0765008500,3/78=0810010000)

4) Orca 5.0 inputs for single-point calculations aiming at energy refinement - (calc 2).

4.1) Singlet

```
! SP R2SCAN D3 6-311++G(d,p) def2-tzvp NormalSCF DEFGRID3 KDIIS NormalPrint

%maxcore 3000

%pal nproc 128 end

%scf
   MaxIter 500
end

%output
   print[p_mos] true
   print[p_basis] 5
end
*xyz 0 1
xyz coordinates
*
```

4.2) Open-shell singlet

```
! UKS SP R2SCAN D3 6-311++G(d,p) def2-tzvp NormalSCF DEFGRID3 KDIIS NormalPrint

%base "2-r2scan-psme-oss-sp"

%maxcore 3000

%pal nproc 128 end

%scf
   MaxIter 500
end

%output
   print[p_mos] true
   print[p_basis] 5
end
*xyz 0 1
xyz coordinates
*
```

4.3) Triplet

```
! SP R2SCAN D3 6-311++G(d,p) def2-tzvp NormalSCF DEFGRID3 KDIIS NormalPrint

%maxcore 3000

%pal nproc 128 end

%scf
   MaxIter 500
end

%output
   print[p_mos] true
   print[p_basis] 5
end
*xyz 0 3
xyz coordinates
*
```

4.3) Quintuplet

```
! SP R2SCAN D3 6-311++G(d,p) def2-tzvp NormalSCF DEFGRID3 KDIIS NormalPrint

%maxcore 3000

%pal nproc 128 end

%scf
   MaxIter 500
end

%output
   print[p_mos] true
   print[p_basis] 5
end

*xyz 0 5
xyz coordinates
*
```

Section S2 – Additional data for Spin-State analysis.

Table S2. Values for $\langle S^2 \rangle$ for the open-shell calculated structures considered in this work. Green-highlighted values are those with $\left|\langle S^2 \rangle\right| > 10\%$ regarding the expected values.

complex		1	2	3	4	5	6	7	8	9	10	11	12
BP86	LS					0.7745							0.7870
	IS	2.3648	2.0247	2.0328	2.0417	3.7878	2.1198	2.0250	2.0237	2.0456	2.0427	2.0425	3.8081
	HS	6.3339	6.0476	6.2892	6.2579	8.7744	6.0884	6.0641	6.0509	6.0272	6.0204	6.0581	8.7797
OPBE	LS					0.8153							0.8124
	IS	2.4862	2.0336	2.0778	2.5151	3.8227	2.2451	2.0450	2.0350	2.1229	2.0721	2.0696	4.0841
	HS	6.4484	6.0638	6.3769	6.4035	8.7781	6.1722	6.1254	6.0711	6.0592	6.0288	6.0987	8.8494
TPSS	LS					0.9111							0.7887
	IS	2.5426	2.0327	2.0524	2.0316	3.7895	2.1428	2.0279	2.0324	2.0449	2.0499	2.0424	3.8068
	HS	6.3738	6.0615	6.3498	6.3094	8.7686	6.0776	6.0665	6.0698	6.0233	6.0179	6.0441	8.7827
M06L	LS					0.7822							1.6362
	IS	2.8882	2.0597	2.4460	2.0310	3.8542	2.5477	2.1330	2.0569	2.1902	2.0872	2.0861	4.4171
	HS	6.5541	6.1239	6.5009	6.5126	8.7700	6.6100	6.1832	6.1276	6.0565	6.0359	6.0999	8.8074
r^{2}SCAN	LS					0.7765							1.4508
	IS	2.7619	2.0482	2.3551	2.0561	3.8101	2.0512	2.0539	2.0507	2.2961	2.0554	2.0662	3.9566
	HS	6.5045	6.0921	6.0623	6.4219	8.8840	6.1249	6.1215	6.1021	6.0281	6.0226	6.0798	8.7991
B3LYP	LS					0.8570							0.8184
	IS	2.9635	2.0522	2.5376	2.1162	3.8182	2.3010	2.0738	2.0543	2.1073	2.0551	2.0402	4.4358
	HS	6.4448	6.1248	6.5064	6.4044	8.7652	6.0535	6.0979	6.1176	6.0166	6.0168	6.0208	8.7712
B3LYP*	LS	0.0000	0.0000	0.0000	0.0000	0.8151	0.0000	0.0000	0.0000	0.0000	0.0000	0.0000	1.5502
	IS	2.8168	2.0450	2.0501	2.1022	3.8069	3.0901	2.0684	2.4652	2.0717	2.0513	2.0414	4.2325
	HS	6.4236	6.1002	6.4615	6.3803	8.7654	6.0861	6.0974	6.0975	6.0186	6.0167	6.0222	8.7737
PBE0	LS					0.7749							0.8422
	IS	3.1477	2.0641	2.0875	2.0364	3.8396	2.4150	2.0829	2.0732	2.2382	2.0656	2.0463	3.8798
	HS	6.5712	6.1481	6.5643	6.5108	8.7648	6.0702	6.0879	6.1513	6.0188	6.0193	6.0230	8.7746
TPSSh	LS					0.9029							1.5525
	IS	2.8326	2.0465	2.0934	2.0506	3.8022	2.5730	2.0618	2.0512	2.0531	2.0602	2.0440	4.0219
	HS	6.4585	6.0930	6.4573	6.4169	8.7654	6.0930	6.1041	6.1009	6.0182	6.0177	6.0220	8.7797
M05	LS					0.8387							2.1928
	IS	3.3206	2.0773	2.5942	3.1201	3.8803	3.2659	2.1483	2.1316	2.2121	2.0661	2.0630	4.5886
	HS	6.6461	6.1319	6.5591	6.6214	8.7708	6.8799	6.1775	6.1803	6.0286	6.0262	6.0505	8.7909
M06	LS					0.7976							1.9888
	IS	3.2934	2.0697	2.0702	2.1338	3.8630	2.1914	2.1222	2.0867	2.0719	2.0702	2.0599	4.5004
	HS	6.5760	6.1625	6.5627	6.5481	8.7703	6.1576	6.1549	6.1454	6.0306	6.0394	6.0445	8.7921
ω-B97XD	LS					0.8868							1.0360
	IS	2.0722	2.6456	2.0160	2.0148	3.8441	2.2150	2.0525	2.0721	2.1393	2.0373	2.0301	4.5433
	HS	6.5870	6.1151	6.5323	6.3168	9.1908	6.0694	6.1184	6.1294	6.0150	6.0145	6.0180	8.7765
r^{2}SCAN	LS					0.7765							1.4508
	IS	2.7619	2.0482	2.3551	2.0561	3.8101	2.0512	2.0539	2.0507	2.2961	2.0554	2.0662	3.9566
	HS	6.5045	6.0921	6.0623	6.4219	8.8840	6.1249	6.1215	6.1021	6.0281	6.0226	6.0798	8.7991

s stands for singlet, *oss* for open-shell singlet, *t* for triplet, and *q* for quintuplet. In the case of complexes with odd number of electrons (**5** and **12**) s, t, and *q* stands for the doublet, quadruplet, and sextuplet, respectively.

For most of the species reported in this work, the spin-contamination of the open-shell calculations was smaller than 10%. as is shown in **Table S2**.

In the case of spin-contaminated structures we performed correction by the following equation, in order to obtain the spin-corrected energies $E_{spin-corrected}$:

$$E_{spin-corrected} = \frac{E_S - a \cdot (E_{(S+1)})}{1 - a}$$

$$a = \frac{\langle S_S^2 \rangle - S(S+1)}{\langle S_{S+1}^2 \rangle \; - S(S+1)}$$

Where E_S and $\langle S_S^2 \rangle$ are the electronic energy and square total spin angular momentum of the S spin-state (i.e. triplet state) obtained by means of an unrestricted calculation. $E_{(S+1)}$and $\langle S_{S+1}^2 \rangle$ are the electronic energy and square total spin angular momentum obtained for the (S+1) spin-state computed with the same level of theory and at the geometry of the S spin-state. $S(S+1)$ is the theoretical S^2 of the spin-state to be corrected. The energies were obtained at XCF/6-311++G(d.p)/SMD single point calculation on the optimized structure at XCF/6-31+G(d.p) level of theory. XCF stands for a given exchange-correlation functional. The corrected energies are summarized in **Table S3**.

Table S3. Energy values before and after spin contamination corrections.

species		BP86	OPBE	TPSS	M06L	B3LYP	B3LYP*	PBE0	TPSSh	M05	M06	ω-B97XD	r^2-SCAN
**1-T**	before	-17.83	-27.92	-32.08	-32.08	-25.97	-25.97	-37.71	-24.16	-51.30	-39.58		-30.82
	after	-17.27	-26.45	-27.04	-27.04	-22.87	-22.87	-32.01	-21.37	-42.77	-30.19		-28.66
**1-Q**	before									-73.36			
	after									-74.71			
**2-T**	before											11.32	
	after											9.46	
**3-T**	before			11.26	11.26					-2.79			44.94
	after			12.29	12.29					-0.28			43.57
**4-T**	before		20.38							-7.44			
	after		19.49							-2.40			
**4-Q**	before									-24.70			
	after									-26.08			
**5-4**	before								-11.07	-22.53		-4.45	
	after								-11.65	-23.20		-4.66	
**5-6**	before								1.44	-25.07		18.23	
	after								0.85	-25.75		18.02	
**6-T**	before		-6.00	-11.45	-11.45			-12.50	-10.98	-23.45		0.30	
	after		-6.51	-11.57	-11.57			-12.47	-12.24	-19.99		1.19	
**6-Q**	before			-12.71	-12.71	-15.02	-15.02			-35.03			
	after			-12.66	-12.66	-18.26	-18.26			-36.27			
**9-T**	before					2.65	2.65	-21.51		-21.65	-22.09		-13.76
	after					1.01	1.01	-20.25		-19.96	-21.69		-12.33
**12-4**	before							-16.41	-6.66	-19.34	-16.26	-32.89	-7.00
	after							-16.78	-7.59	-30.30	-25.57	-36.33	-7.75
**12-6**	before			-3.43	-3.43			-25.72	2.58	-13.62	-9.47	-26.72	5.26
	after			-11.16	-11.16			-26.09	1.65	-23.38	-17.42	-29.18	4.50

[a]T - triplete, Q- quintuplet, OSS-open-shell singlet, 4-Quadruplet, 6-Sextuplet.

Table S4 summarizes our observations about the number of errors made by various exchange-correlation functionals (XCFs) in reproducing the correct ground spin state of iron complexes, both with and without effective core potentials (ECP). The system where a given XCF does not reproduce the experimental spin-state is in bold-italic.

Table S4. Energy values using and not using ECP SDD for iron.

**complex**		**1**		**2**		**3**		**4**		**5**		**6**	
Exp. spin (S)[a]		2		0		0		0		3/2		1	
		**ECP**	**NoECP**	**ECP**	**NoECP**	**ECP**	**NoECP**	**ECP**	**NoECP**	**ECP**	**NoECP**	**ECP**	**NoECP**
BP86	LS	0.00	0.00[f]	0.00	0.00	0.00	0.00	0.00	0.00	0.00	0.00	0.00	0.00
	IS	-19.59	-17.27	19.78	22.45	18.00	20.39	21.40	24.25	-6.67	-3.77	-3.39	-1.24
	HS	-31.99	-26.90	41.57	48.27	23.63	31.24	27.54	36.11	9.62	15.61	3.15	7.66
OPBE	LS	0.00	0.00	0.00	0.00	0.00	0.00	0.00	0.00	0.00	0.00	0.00	0.00
	IS	-30.45	-26.45	21.53	23.93	15.83	18.32	25.24	19.49	-16.39	-13.06	-8.81	-6.51
	HS	-48.58	-42.48	43.04	49.16	9.23	17.18	14.18	23.68	-7.33	-1.07	-6.02	-0.60
TPSS	LS	0.00	0.00	0.00	0.00	0.00	0.00	0.00	0.00	0.00	0.00	0.00	0.00
	IS	-22.75	-16.55	15.96	19.31	11.68	15.73	12.88	15.63	-10.35	-3.65	-8.90	-3.97
	HS	-40.40	-28.66	32.30	42.29	12.14	26.01	18.64	33.78	1.33	14.04	-3.21	4.52
M06-L	LS	0.00	0.00	0.00	0.00	0.00	0.00	0.00	0.00	0.00	0.00	0.00	0.00
	IS	-28.47	-27.04	14.26	14.88	9.89	12.29	18.00	19.19	-11.96	-4.86	-12.83	-12.66
	HS	-54.12	-56.29	22.72	23.35	2.73	0.40	4.00	2.07	-2.42	-3.70	-17.43	**-18.79**
r^2-SCAN	LS	0.00	0.00	0.00	0.00	0.00	0.00	0.00	0.00		0.00	0.00	0.00
	IS	-27.17	-28.66	11.72	13.68	12.57	43.57		21.43		-12.15	-1.25	-0.78
	HS	-44.65	-42.34	34.24	35.37	31.73	58.41	15.25	16.79		9.00	2.30	1.84
B3LYP	LS	0.00	0.00	0.00	0.00	0.00	0.00	0.00	0.00	0.00	0.00	0.00	0.00
	IS	-25.81	-24.73	8.27	10.56	1.27	6.51	14.28	15.83	-13.95	-12.29	-12.37	-9.95
	HS	-48.58	-45.42	12.81	18.40	20.64	3.13	0.46	5.90	-8.30	-4.35	-12.23	-9.02
B3LYP*	LS	0.00	0.00	0.00	0.00	0.00	0.00	0.00	0.00	0.00	0.00	0.00	0.00
	IS	-24.37	-22.87	11.16	13.52	4.88	20.08	15.13	16.81	-12.48	-10.27	-17.56	-18.26
	HS	-44.68	-40.82	18.87	24.70	25.68	9.67	5.40	11.37	-2.94	1.79	-5.86	-5.86
PBE0	LS	0.00	0.00	0.00	0.00	0.00	0.00	0.00	0.00	0.00	0.00	0.00	0.00
	IS	-36.11	-32.01	4.98	7.59	-3.70	15.18	11.89	23.27	-22.52	-19.36	-16.29	-12.47
	HS	-61.38	-55.37	8.58	15.23	15.05	***-5.53***	-5.60	2.47	-21.21	-15.58	-18.49	***-14.33***
TPSSh	LS	0.00	0.00	0.00	0.00	0.00	0.00	0.00	0.00	0.00	0.00	0.00	0.00
	IS	-28.17	-21.37	10.99	14.45	16.17	19.77	10.31	13.45	-16.82	-11.65	-18.78	-12.24
	HS	-49.91	-38.17	21.71	31.66	23.30	13.67	8.17	22.69	-9.73	0.85	-11.38	-3.71
M05	LS	0.00	0.00	0.00	0.00	0.00	0.00	0.00	0.00	0.00	0.00	0.00	0.00
	IS	-53.70	-42.77	8.93	9.80	-10.35	-0.28	11.86	-2.40	-29.92	-23.20	-27.83	-19.99
	HS	-78.94	-74.71	4.81	9.57	2.02	***-21.90***	-30.76	***-26.08***	-30.14	***-25.75***	-25.67	***-36.27***
M06	LS	0.00	0.00	0.00	0.00	0.00	0.00	0.00	0.00	0.00	0.00	0.00	0.00
	IS	-41.45	-30.19	7.53	8.65	-10.09	21.07	0.12	13.23	-29.32	-19.15	-29.88	-5.76
	HS	-77.62	-64.90	1.55	9.54	3.46	***-16.26***	-24.53	***-12.42***	-28.10	-17.40	-25.87	13.34
ω-B97XD	LS	0.00	0.00	0.00	0.00	0.00	0.00	0.00	0.00	0.00	0.00	0.00	0.00
	IS	-2.88	6.52	14.79	9.46	20.15	21.89	26.38	27.53	-15.65	-4.66	-0.44	1.19
	HS	-46.18	-43.16	13.44	20.50	-6.04	26.66	99.26	23.94	14.38	18.02	-22.86	***-18.01***

Continuation of Table S4.

complex		7		8		9		10		11		12	
Exp. spin (S)[a]		0		0		2		2		1		3/2	
		ECP	NoECP	ECP	NoECP	ECP	NoECP	ECP	NoECP	ECP	NoECP	ECP	NoECP
BP86	LS	0.00	0.00	0.00	0.00	0.00	0.00	0.00	0.00	0.00	0.00	0.00	***0.00***[g]
	IS	11.67	14.01	12.10	14.10	-17.53	-12.63	-31.55	-28.35	-9.70	-7.35	0.91	5.60
	HS	20.64	27.01	27.42	33.78	-27.14	-19.30	-33.43	-26.02	8.83	17.23	12.96	21.35
OPBE	LS	0.00	0.00	0.00	0.00	0.00	0.00	0.00	0.00	0.00	0.00	0.00	***0.00***
	IS	11.46	14.22	15.05	16.77	-20.56	-16.61	-36.37	***-32.87***	-10.57	-7.53	-5.31	0.14
	HS	19.70	26.96	28.71	34.96	-35.31	-27.20	-30.09	-22.77	4.49	13.33	3.31	11.53
TPSS	LS	0.00	0.00	0.00	0.00	0.00	0.00	0.00	0.00	0.00	0.00	0.00	***0.00***
	IS	11.34	14.93	12.49	15.23	-19.31	-13.81	-38.14	***-31.71***	-13.34	-8.81	-5.85	2.31
	HS	13.61	23.93	21.10	31.40	-33.17	-21.99	-44.72	-31.54	-3.12	11.04	3.18	17.01
M06-L	LS	0.00	0.00	0.00	0.00	0.00	0.00	0.00	0.00	0.00	0.00	0.00	0.00
	IS	8.86	9.62	9.92	10.75	-17.66	-14.77	-29.40	-26.37	-16.02	-15.45	-18.07	-21.29
	HS	7.57	9.37	12.27	13.36	-34.33	-33.03	-46.82	-43.82	-14.20	-12.86	-7.10	-6.34
r^2-SCAN	LS	0.00	0.00	0.00	0.00	0.00	0.00	0.00	0.00	0.00	0.00	0.00	0.00
	IS	14.77	7.07	9.94	10.58	-13.62	-12.33	-22.75	-24.04	-14.17	-13.69	-16.73	-7.75
	HS	25.03	20.62	24.19	25.02	-35.17	-31.46	-32.92	-34.02	19.09	20.42	-7.90	4.50
B3LYP	LS	0.00	0.00	0.00	0.00	0.00	0.00	0.00	0.00	0.00	0.00	0.00	0.00
	IS	0.92	3.54	6.92	8.50	-17.58	-16.52	-40.50	-39.28	-15.79	-15.06	-31.70	-33.21
	HS	-0.57	6.00	3.76	8.40	-32.08	-28.81	-58.15	-53.71	-13.81	-8.72	-25.18	-15.35
B3LYP*	LS	0.00	0.00	0.00	0.00	0.00	0.00	0.00	0.00	0.00	0.00	0.00	0.00
	IS	4.03	6.67	-3.30	1.01	-17.51	-16.33	-38.08	-36.61	-13.87	-12.96	-17.28	-18.74
	HS	-2.06	2.96	8.31	13.28	-36.71	-33.47	-53.24	-48.36	-7.93	-2.32	-9.46	-3.34
PBE0	LS	0.00	0.00	0.00	0.00	0.00	0.00	0.00	0.00	0.00	0.00	0.00	0.00
	IS	-1.72	2.13	4.15	6.22	-23.9	-20.25	-45.93	-44.65	-20.01	-17.41	-28.22	-16.78
	HS	-6.17	2.65	0.35	6.56	-51.25	-45.36	-73.06	-66.05	-21.75	-13.93	-19.97	***-26.09***
TPSSh	LS	0.00	0.00	0.00	0.00	0.00	0.00	0.00	0.00	0.00	0.00	0.00	0.00
	IS	5.99	11.43	8.17	11.01	-23.11	-17.57	-43.90	-37.83	-16.32	-11.69	-16.00	-7.59
	HS	3.13	13.93	11.80	21.90	-35.45	-24.08	-56.81	-44.15	-11.00	2.65	-10.62	1.65
M05	LS	0.00	0.00	0.00	0.00	0.00	0.00	0.00	0.00	0.00	0.00	0.00	0.00
	IS	-8.40	-4.25	3.35	4.64	-26.27	-19.96	-43.18	-39.17	-31.26	-25.90	-42.82	-30.30
	HS	-16.00	***-7.87***	-14.35	***-8.30***	-64.58	-55.92	-71.73	-62.52	-36.32	***-28.18***	-36.26	-23.38
M06	LS	0.00	0.00	0.00	0.00	0.00	0.00	0.00	0.00	0.00	0.00	0.00	0.00
	IS	-6.41	-0.89	7.07	8.88	-28.40	-21.69	-44.52	-38.66	-26.53	-19.40	-36.40	-25.57
	HS	-11.87	***-1.25***	-6.12	3.28	-63.06	-50.07	-69.40	-61.61	-35.41	***-20.29***	-29.00	-17.42
ω-B97XD	LS	0.00	0.00	0.00	0.00	0.00	0.00	0.00	0.00	0.00	0.00	0.00	0.00
	IS	-2.08	2.07	4.54	5.91	-18.02	-17.52	-34.42	-32.42	-17.11	-15.79	-41.77	-36.33
	HS	29.77	52.62	2.66	8.42	-44.13	-39.84	-57.91	-53.34	-18.17	-11.21	-37.01	-29.18

TPSS (with ECP), TPSSh (with ECP), and B3LYP/B3LYP* (without ECP) showed perfect performance with zero errors. In contrast, BP86 exhibited 12 errors both with and without ECP, while M05 showed 6 errors without ECP and 6 errors with ECP. M06 made 5 errors without ECP and 5 with ECP. XCF such as OPBE had fewer errors (1 without ECP, 2 with ECP), and PBE0 (the second occurrence) showed improved accuracy with ECP, reducing from 3 errors (no ECP) to 5 (with ECP). ωB97XD performed better without ECP (1 error)

than with ECP (4 errors). These results highlight that while some functionals maintain consistent performance regardless of ECP (e.g., M06-L with 1 error in both cases), others are more sensitive to its inclusion. Overall, considering the ECP we have 29 errors while not using the ECP we have 19 errors.

Section S3 – Additional data for RMSD analysis.

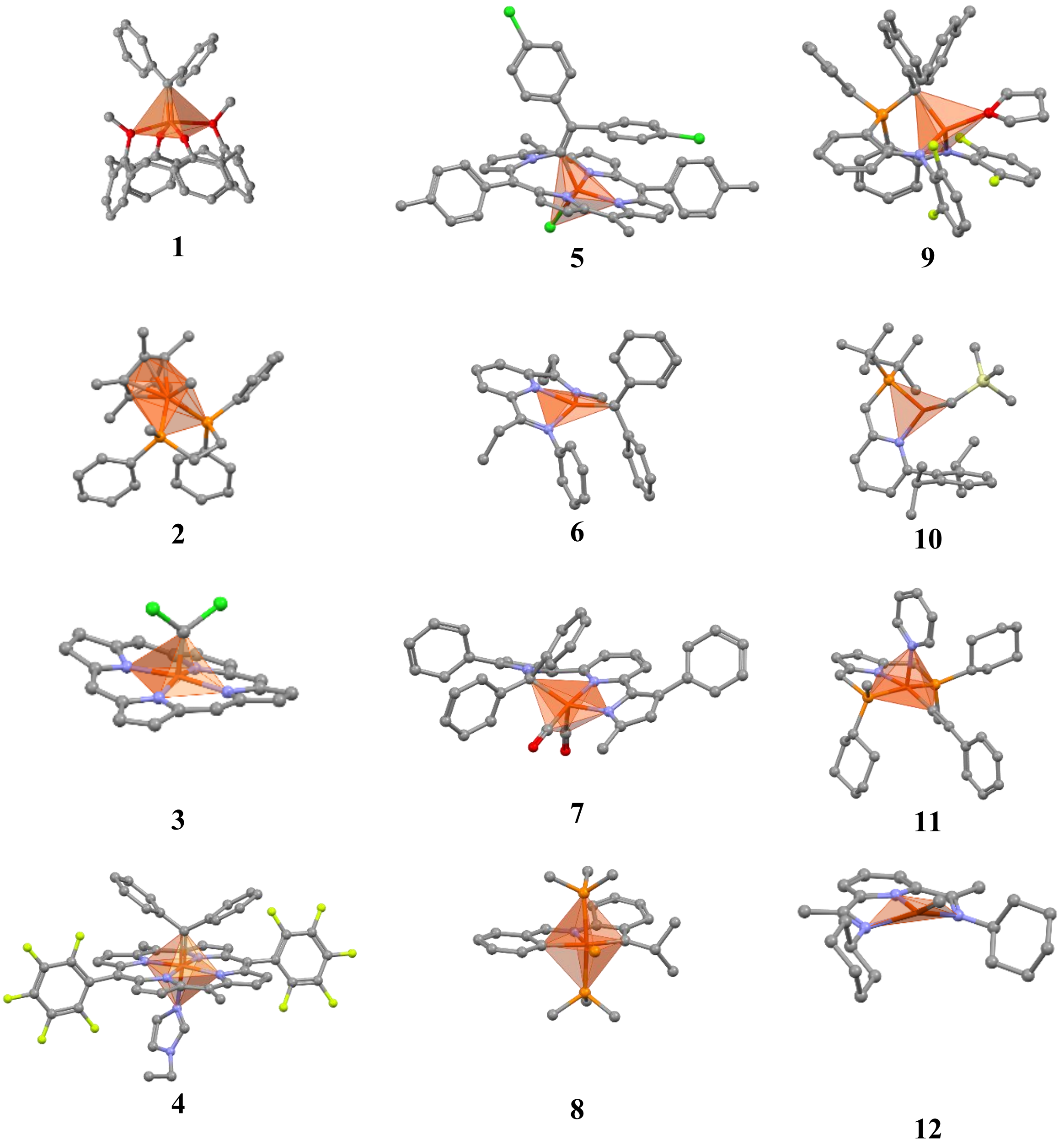

Figure S1. Definition of the first coordination sphere in complexes **1-12**.

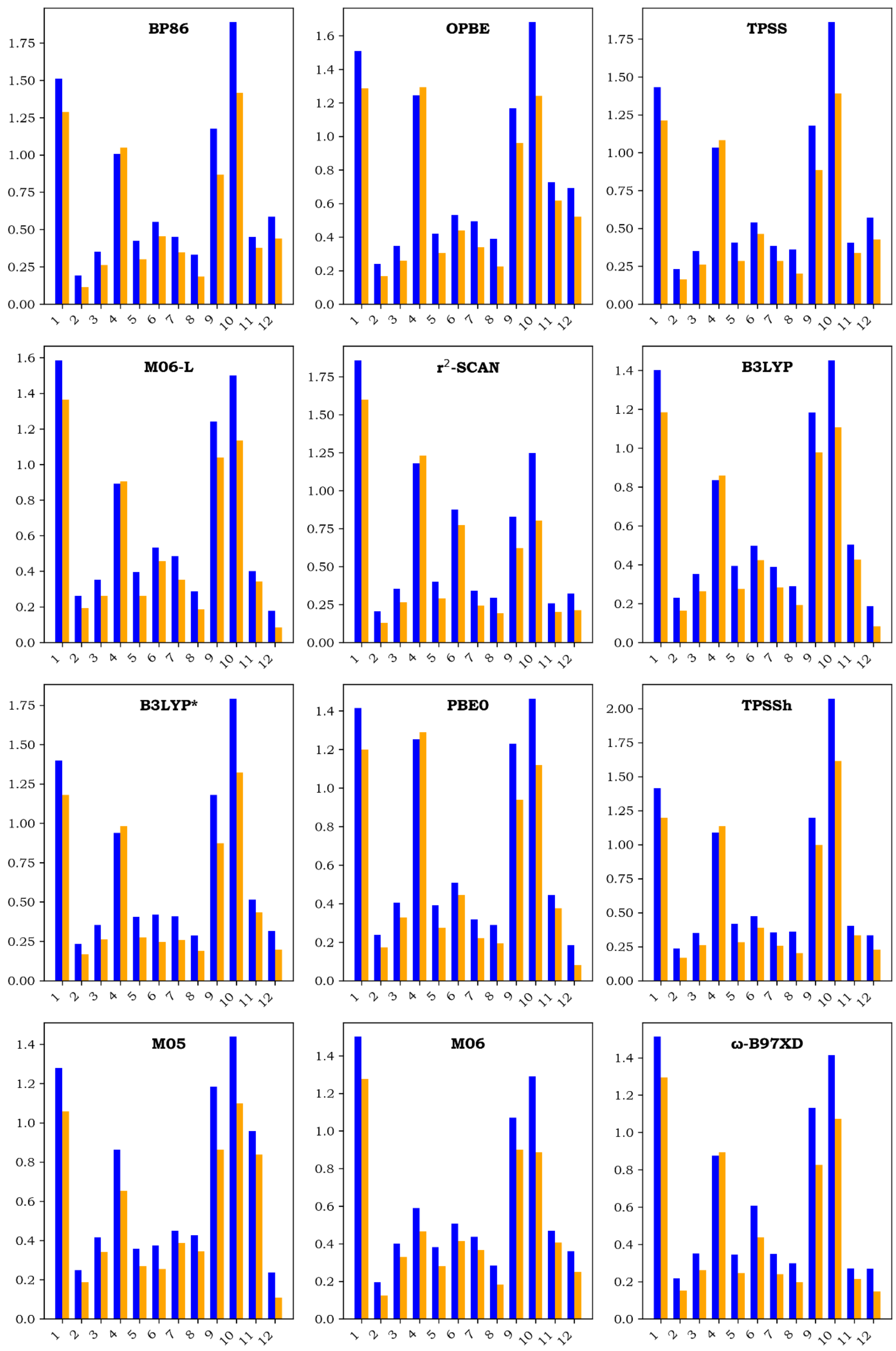

Figure S2. Comparison of the RMSD, against the experimental x-ray structure, when considering all the atoms (blue) and considering only the heavy atoms (orange)

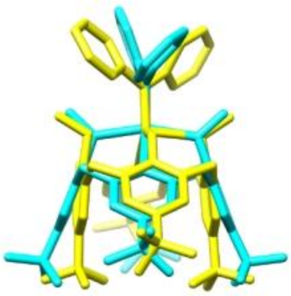

1 quintet

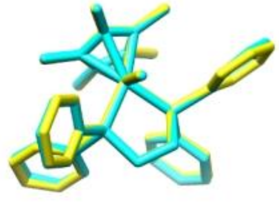

2 singlet

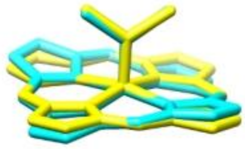

3 singlet

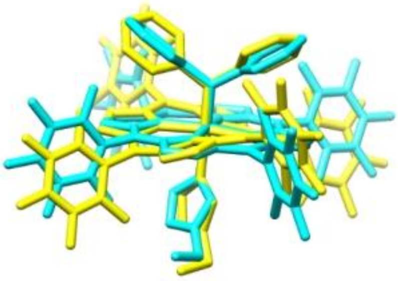

4 singlet

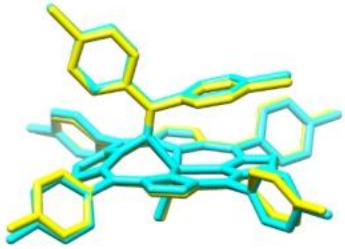

5 quartet

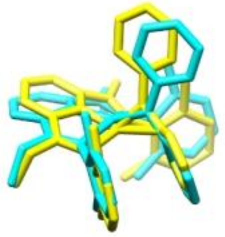

6 quintet

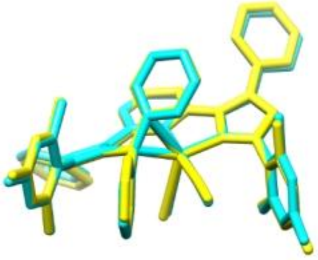

7 singlet

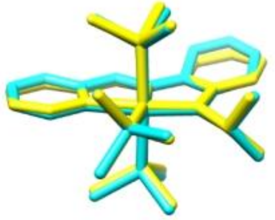

8 singlet

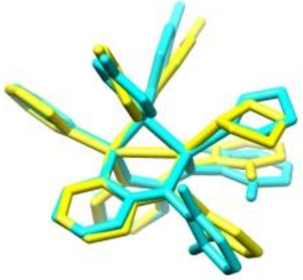

9 quintet

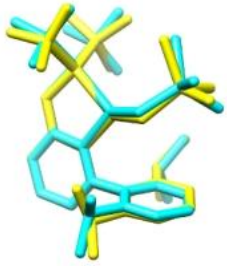

10 quintet

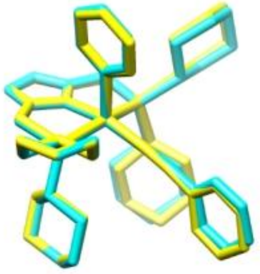

11 triplet

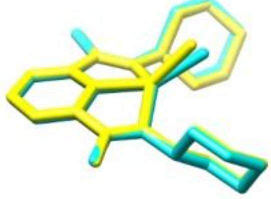

12 quartet

Figure S3. Superposition of the x-ray experimental (blue) structure and the optimized structure with r^2-SCAN (yellow).

Section S4 – Additional data used to kinetic analysis.

BP86

system	Exp.ΔΔG	xTBΔΔG	|exp-calc|	14p	13p	13pNoI	10p
I	-2.727	9.400	12.127	12.127			
II	-1.216	-0.870	0.346	0.346	0.346	0.346	0.346
III	-0.916	7.160	8.076	8.076	8.076	8.076	
IV	0.000	-0.120	0.120	0.120	0.120	0.120	0.120
V	-1.563	-1.150	0.413	0.413	0.413	0.413	0.413
VI	-0.410	-0.360	0.050	0.050	0.050	0.050	0.050
VII	-1.774	-1.250	0.524	0.524	0.524	0.524	0.524
VIII	-1.519	-0.810	0.709	0.709	0.709	0.709	0.709
IX	-1.231	-1.570	0.339	0.339	0.339	0.339	0.339
X	-0.410	-0.750	0.340	0.340	0.340	0.340	0.340
XI	-1.369	0.640	2.009	2.009	2.009	2.009	
XII	-1.177	-1.780	0.603	0.603	0.603	0.603	0.603
XIII	-0.439	1.280	1.719	1.719	1.719	1.719	
XIV	-2.305	-1.680	0.625	0.625	0.625	0.625	0.625
MAE				2.000	1.221	1.221	0.407

OPBE

system	Exp.ΔΔG	OPBEΔΔG	|exp-calc|	14p	13p	13pNoI	10p
I	-2.727	-1.190	1.537	1.537	1.537		
II	-1.216	0.190	1.406	1.406	1.406	1.406	
III	-0.916	0.260	1.176	1.176	1.176	1.176	1.176
IV	0.000	-0.540	0.540	0.540	0.540	0.540	0.540
V	-1.563	-0.490	1.073	1.073	1.073	1.073	1.073
VI	-0.410	0.340	0.750	0.750	0.750	0.750	0.750
VII	-1.774	-0.680	1.094	1.094	1.094	1.094	1.094
VIII	-1.519	-0.880	0.639	0.639	0.639	0.639	0.639
IX	-1.231	-1.000	0.231	0.231	0.231	0.231	0.231
X	-0.410	-0.740	0.330	0.330	0.330	0.330	0.330
XI	-1.369	-1.920	0.551	0.551	0.551	0.551	0.551
XII	-1.177	-0.550	0.627	0.627	0.627	0.627	0.627
XIII	-0.439	-2.530	2.091	2.091	2.091	2.091	
XIV	-2.305	0.410	2.715	2.715		2.715	
MAE				1.054	0.927	1.017	0.701

TPSS

system	Exp.ΔΔG	TPSSΔΔG	|exp-calc|	14p	13p	13pNoI	10p
I	-2.727	-1.530	1.197	1.197	1.197		1.197
II	-1.216	-1.070	0.146	0.146	0.146	0.146	0.146
III	-0.916	-1.520	0.604	0.604	0.604	0.604	0.604
IV	0.000	5.010	5.010	5.010		0.000	
V	-1.563	1.080	2.643	2.643	2.643	2.643	
VI	-0.410	-0.870	0.460	0.460	0.460	0.460	0.460
VII	-1.774	-1.190	0.584	0.584	0.584	0.584	0.584
VIII	-1.519	-1.100	0.419	0.419	0.419	0.419	0.419
IX	-1.231	-1.010	0.221	0.221	0.221	0.221	0.221
X	-0.410	0.690	1.100	1.100	1.100	1.100	1.100
XI	-1.369	-1.170	0.199	0.199	0.199	0.199	0.199
XII	-1.177	-1.620	0.443	0.443	0.443	0.443	0.443
XIII	-0.439	3.570	4.009	4.009	4.009	4.009	
XIV	-2.305	1.120	3.425	3.425	3.425	3.425	
MAE				1.461	1.188	1.096	0.537

M06-L

system	Exp.ΔΔG	M06LΔΔG	|exp-calc|	14p	13p	13pNoI	10p
I	-2.727	-1.850	0.877	0.877	0.877		0.877
II	-1.216	-0.660	0.556	0.556	0.556	0.556	0.556
III	-0.916	-1.230	0.314	0.314	0.314	0.314	0.314
IV	0.000	-1.690	1.690	1.690	1.690	1.690	1.690
V	-1.563	-2.930	1.367	1.367	1.367	1.367	1.367
VI	-0.410	-0.010	0.400	0.400	0.400	0.400	0.400
VII	-1.774	-1.860	0.086	0.086	0.086	0.086	0.086
VIII	-1.519	-2.180	0.661	0.661	0.661	0.661	0.661
IX	-1.231	-3.190	1.959	1.959	1.959	1.959	
X	-0.410	-4.090	3.680	3.680	3.680	3.680	
XI	-1.369	3.830	5.199	5.199	5.199	5.199	
XII	-1.177	-1.680	0.503	0.503	0.503	0.503	0.503
XIII	-0.439	0.430	0.869	0.869	0.869	0.869	0.869
XIV	-2.305	-8.220	5.915	5.915		0.000	
MAE				1.720	1.397	1.330	0.732

r^2-SCAN

system	Exp.ΔΔG	r^2-SCAN ΔΔG	|exp-calc|	14p	13p	13pNoI	10p
I	-2.727	-2.650	0.077	0.077	0.077		0.077
II	-1.216	-0.700	0.516	0.516	0.516	0.516	0.516
III	-0.916	-1.580	0.664	0.664	0.664	0.664	0.664
IV	0.000	-8.290	8.290	8.290		0.000	
V	-1.563	-2.010	0.447	0.447	0.447	0.447	0.447
VI	-0.410	-0.160	0.250	0.250	0.250	0.250	0.250
VII	-1.774	-1.550	0.224	0.224	0.224	0.224	0.224
VIII	-1.519	-1.420	0.099	0.099	0.099	0.099	0.099
IX	-1.231	-1.230	0.001	0.001	0.001	0.001	0.001
X	-0.410	-0.200	0.210	0.210	0.210	0.210	0.210
XI	-1.369	-1.740	0.371	0.371	0.371	0.371	0.371
XII	-1.177	-0.330	0.847	0.847	0.847	0.847	
XIII	-0.439	1.850	2.289	2.289	2.289	2.289	
XIV	-2.305	0.340	2.645	2.645	2.645	2.645	
MAE				1.209	0.665	0.659	0.286

B3LYP

system	Exp.ΔΔG	xTBΔΔG	|exp-calc|	14p	13p	13pNoI	10p
I	-2.727	-1.330	1.397	1.397	1.397		1.397
II	-1.216	-0.880	0.336	0.336	0.336	0.336	0.336
III	-0.916	-0.270	0.646	0.646	0.646	0.646	0.646
IV	0.000	-0.040	0.040	0.040	0.040	0.040	0.040
V	-1.563	-1.160	0.403	0.403	0.403	0.403	0.403
VI	-0.410	-1.400	0.990	0.990	0.990	0.990	0.990
VII	-1.774	-0.270	1.504	1.504	1.504	1.504	1.504
VIII	-1.519	0.010	1.529	1.529	1.529	1.529	1.529
IX	-1.231	-0.990	0.241	0.241	0.241	0.241	0.241
X	-0.410	1.130	1.540	1.540	1.540	1.540	
XI	-1.369	1.070	2.439	2.439	2.439	2.439	
XII	-1.177	-1.370	0.193	0.193	0.193	0.193	0.193
XIII	-0.439	3.170	3.609	3.609		0.000	
XIV	-2.305	-0.180	2.125	2.125	2.125	2.125	
MAE				1.214	1.030	0.922	0.728

B3LYP*

system	Exp.ΔΔG	xTBΔΔG	|exp-calc|	14p	13p	13pNoI	10p
**I**	-2.727	-1.670	1.057	1.057	1.057		1.057
**II**	-1.216	-0.450	0.766	0.766	0.766	0.766	0.766
**III**	-0.916	0.080	0.996	0.996	0.996	0.996	0.996
**IV**	0.000	-0.230	0.230	0.230	0.230	0.230	0.230
**V**	-1.563	-0.760	0.803	0.803	0.803	0.803	0.803
**VI**	-0.410	-0.380	0.030	0.030	0.030	0.030	0.030
**VII**	-1.774	-2.130	0.356	0.356	0.356	0.356	0.356
**VIII**	-1.519	-1.530	0.011	0.011	0.011	0.011	0.011
**IX**	-1.231	-0.970	0.261	0.261	0.261	0.261	0.261
**X**	-0.410	-1.720	1.310	1.310	1.310	1.310	
**XI**	-1.369	0.490	1.859	1.859	1.859	1.859	
**XII**	-1.177	-1.550	0.373	0.373	0.373	0.373	0.373
**XIII**	-0.439	2.650	3.089	3.089		3.089	
**XIV**	-2.305	-0.360	1.945	1.945	1.945	1.945	
**MAE**				0.935	0.769	0.925	0.488

PBE0

system	Exp.ΔΔG	xTBΔΔG	|exp-calc|	14p	13p	13pNoI	10p
**I**	-2.727	-0.870	1.857	1.857	1.857		
**II**	-1.216	-1.050	0.166	0.166	0.166	0.166	0.166
**III**	-0.916	-1.190	0.274	0.274	0.274	0.274	0.274
**IV**	0.000	-0.260	0.260	0.260	0.260	0.260	0.260
**V**	-1.563	0.300	1.863	1.863	1.863	1.863	
**VI**	-0.410	-0.190	0.220	0.220	0.220	0.220	0.220
**VII**	-1.774	-1.000	0.774	0.774	0.774	0.774	0.774
**VIII**	-1.519	-1.210	0.309	0.309	0.309	0.309	0.309
**IX**	-1.231	-1.440	0.209	0.209	0.209	0.209	0.209
**X**	-0.410	-1.220	0.810	0.810	0.810	0.810	0.810
**XI**	-1.369	0.800	2.169	2.169	2.169	2.169	
**XII**	-1.177	-1.320	0.143	0.143	0.143	0.143	0.143
**XIII**	-0.439	0.690	1.129	1.129	1.129	1.129	1.129
**XIV**	-2.305	3.690	5.995	5.995		5.995	
**MAE**				1.156	0.783	1.102	0.429

TPSSh

system	Exp.ΔΔG	xTBΔΔG	|exp-calc|	14p	13p	13pNoI	10p
**I**	-2.727	-2.090	0.637	0.637	0.637		0.637
**II**	-1.216	-1.200	0.016	0.016	0.016	0.016	0.016
**III**	-0.916	1.010	1.926	1.926	1.926	1.926	
**IV**	0.000	1.010	1.010	1.010	1.010	1.010	1.010
**V**	-1.563	-0.480	1.083	1.083	1.083	1.083	1.083
**VI**	-0.410	-0.010	0.400	0.400	0.400	0.400	0.400
**VII**	-1.774	-2.320	0.546	0.546	0.546	0.546	0.546
**VIII**	-1.519	-1.990	0.471	0.471	0.471	0.471	0.471
**IX**	-1.231	-2.150	0.919	0.919	0.919	0.919	0.919
**X**	-0.410	-1.590	1.180	1.180	1.180	1.180	1.180
**XI**	-1.369	1.950	3.319	3.319		3.319	
**XII**	-1.177	-1.620	0.443	0.443	0.443	0.443	0.443
**XIII**	-0.439	1.370	1.809	1.809	1.809	1.809	
**XIV**	-2.305	-0.150	2.155	2.155	2.155	2.155	
**MAE**				1.137	0.969	1.175	0.670

M05

system	Exp.ΔΔG	xTBΔΔG	|exp-calc|	14p	13p	13pNoI	10p
**I**	-2.727	-0.550	2.177	2.177	2.177		2.177
**II**	-1.216	0.220	1.436	1.436	1.436	1.436	1.436
**III**	-0.916	-6.560	5.644	5.644		5.644	
**IV**	0.000	0.870	0.870	0.870	0.870	0.870	0.870
**V**	-1.563	-2.490	0.927	0.927	0.927	0.927	0.927
**VI**	-0.410	1.860	2.270	2.270	2.270	2.270	
**VII**	-1.774	-0.520	1.254	1.254	1.254	1.254	1.254
**VIII**	-1.519	-1.080	0.439	0.439	0.439	0.439	0.439
**IX**	-1.231	-1.220	0.011	0.011	0.011	0.011	0.011
**X**	-0.410	1.360	1.770	1.770	1.770	1.770	1.770
**XI**	-1.369	0.220	1.589	1.589	1.589	1.589	1.589
**XII**	-1.177	-0.890	0.287	0.287	0.287	0.287	0.287
**XIII**	-0.439	2.440	2.879	2.879	2.879	2.879	
**XIV**	-2.305	3.190	5.495	5.495	5.495	5.495	
**MAE**				1.932	1.647	1.913	1.076

M06

system	Exp.ΔΔG	xTBΔΔG	|exp-calc|	14p	13p	13pNoI	10p
I	-2.727	0.310	3.037	3.037	3.037		
II	-1.216	-0.240	0.976	0.976	0.976	0.976	0.976
III	-0.916	0.680	1.596	1.596	1.596	1.596	
IV	0.000	-0.890	0.890	0.890	0.890	0.890	0.890
V	-1.563	-0.650	0.913	0.913	0.913	0.913	0.913
VI	-0.410	-0.530	0.120	0.120	0.120	0.120	0.120
VII	-1.774	0.260	2.034	2.034	2.034	2.034	
VIII	-1.519	-2.740	1.221	1.221	1.221	1.221	1.221
IX	-1.231	-1.480	0.249	0.249	0.249	0.249	0.249
X	-0.410	-3.920	3.510	3.510		3.510	
XI	-1.369	0.120	1.489	1.489	1.489	1.489	1.489
XII	-1.177	-1.020	0.157	0.157	0.157	0.157	0.157
XIII	-0.439	-0.380	0.059	0.059	0.059	0.059	0.059
XIV	-2.305	-2.300	0.005	0.005	0.005	0.005	0.005
MAE				1.161	0.980	1.017	0.608

ω-B97XD

system	Exp.ΔΔG	xTBΔΔG	|exp-calc|	14p	13p	13pNoI	10p
I	-2.727	-2.410	0.317	0.317	0.317		0.317
II	-1.216	0.360	1.576	1.576	1.576	1.576	1.576
III	-0.916	-1.760	0.844	0.844	0.844	0.844	0.844
IV	0.000	-0.930	0.930	0.930	0.930	0.930	0.930
V	-1.563	-0.630	0.933	0.933	0.933	0.933	0.933
VI	-0.410	-2.950	2.540	2.540	2.540	2.540	2.540
VII	-1.774	2.430	4.204	4.204	4.204	4.204	
VIII	-1.519	-3.920	2.401	2.401	2.401	2.401	2.401
IX	-1.231	-1.940	0.709	0.709	0.709	0.709	0.709
X	-0.410	-1.550	1.140	1.140	1.140	1.140	1.140
XI	-1.369	-8.670	7.301	7.301		7.301	
XII	-1.177	-2.740	1.563	1.563	1.563	1.563	1.563
XIII	-0.439	2.970	3.409	3.409	3.409	3.409	
XIV	-2.305	-5.160	2.855	2.855	2.855	2.855	
MAE				2.194	1.802	2.339	1.295

Section S5 – Performance of GFN2-xTB

Upon the suggestion of one of the reviewers we performed the calculations we did for each XCF with GFN2-xTB, since it is a semiempirical inexpensive method, and a good performance would be of valuable value. **Table S5** shows the relative energy of the different spin-states. In it we can the method failed to reproduce the experimental spin-state in seven cases, as much as the case for M05 (see **Table 1**), the worst XCF performance.

Table S5. Calculated spin-state energies (kcal·mol^{-1}) with exchange-correlation functionals for the different Fe^{II} complexes using GFN2-xTB.

System	1	2	3	4	5	6	7	8	9	10	11	12
**Exp.**	2	0	0	0	3/2	1	0	0	2	2	1	3/2
**Singlet**	**0.00**	0.00	**0.00**	**0.00**	**0.00**	**0.00**	**0.00**	**0.00**	**0.00**	0.00	**0.00**	**0.00**
**Triplet**	3.82	**-2.79**	26.29	19.87	18.30	6.92	23.28	20.79	8.05	**-0.15**	8.93	26.32
**Quintet**	23.07	33.05	58.51	50.28	55.88	30.28	56.97	62.50	33.13	31.32	60.91	51.54

Table S6 summarizes the RMSD, as analyzed in **Figure 1** and **2**. The summation of all heavy atoms, without taking the hydrogens into consideration, lead to a value of 7.8107 Å, bigger than all the XCFs. Regarding only the polyhedron of the first coordination sphere, we have the value of 3.4332 Å, away bigger than the worst performing XCF M05 (see **Figure 2**), with a value of 2.605 Å.

Table S6. RMSD (Å) of the most stable spin-state of each complex for each XCF, for the all the atoms, hydrogens atoms excluded.

Complex	1	2	3	4	5	6	7	8	9	10	11	12	Sum (Σ)
**All atoms**	1.9086	0.2476	0.3705	0.7282	0.4006	0.9920	0.4929	0.3387	2.1758	1.4208	0.4967	0.7234	9.8018
**Heavy atoms**	1.6643	0.1824	0.2762	0.7343	0.2991	0.8481	0.3975	0.2277	1.8063	1.0597	0.4319	0.5429	7.8107
**polyhedron**	0.2216	0.0576	0.0483	0.0447	0.1394	0.8860	0.0576	0.1212	0.5480	0.2355	0.0688	0.0751	3.4332

Regarding kinetics reproduction, **Table S7** shows the stereoselectivity that GFN2-xTB yields. Only in cases II and VI does the method produce values approaching experimental results. Regarding the MAE, xTB shows variable performance, ranking 7th in 13pNo1 but dropping to 9th-10th in other sets. While it consistently beats the worst DFT functionals (M05/ωB97X-D), its accuracy remains moderate overall.

Overall, the analysis reveals GFN2-xTB's significant limitations for iron-carbene systems, since it fails to reproduce the geometry, spin-states and kinetics considered in this work. These results demonstrate that GFN2-xTB fails to properly describe three critical aspects: (1) system geometries, (2) spin-state energetics. In the kinetical analysis it performs best the some XCF, but still in the lower part of the herein considered

methods. We therefore conclude that tts speed/accuracy balance makes it useful for preliminary studies, though DFT (especially r²SCAN, B3LYP*) is preferable for final benchmarks.

Table S7. xTB data for stereoselectivity analysis

system	Exp.ΔΔG	xTBΔΔG	|exp-calc|	14p	13p	13pNoI	10p
**I**	-2.727	0.638	3.365	3.365	3.365		
**II**	-1.216	-1.234	0.018	0.018	0.018	0.018	0.018
**III**	-0.916	0.830	1.746	1.746	1.746	1.746	1.746
**IV**	0.000	15.387	1.539	1.539	1.539	1.539	1.539
**V**	-1.563	-0.057	1.505	1.505	1.505	1.505	1.505
**VI**	-0.410	-0.480	0.069	0.069	0.069	0.069	0.069
**VII**	-1.774	0.013	1.787	1.787	1.787	1.787	
**VIII**	-1.519	-0.298	1.221	1.221	1.221	1.221	1.221
**IX**	-1.231	22.166	3.448	3.448		0.000	
**X**	-0.410	17.028	2.113	2.113	2.113	2.113	
**XI**	-1.369	-0.141	1.228	1.228	1.228	1.228	1.228
**XII**	-1.177	-17.392	0.562	0.562	0.562	0.562	0.562
**XIII**	-0.439	-0.141	0.298	0.298	0.298	0.298	0.298
**XIV**	-2.305	-18.249	0.480	0.480	0.480	0.480	0.480
**MAE**				1.384	1.226	0.967	0.867

Section S8 - Influence of the solvent in the spin-state energetic.

Table S8 – Comparison of solvent influence. Energies in kcal.mol^{-1}.NS stands for no-solvent (calculated with double-zeta basis set without including solvent via SMD method) and WS stands for with solvent (calculated with triple zeta basis set and solvent via SMD).

Complex		1		2		3		4		5		6	
Exp. Spin (S)		2		0		0		0		3/2		1	
		NS	WS	NS	WS	NS	WS	NS	WS	NS	WS	NS	WS
	LS	0.00	0.00	0.00	0.00	0.00	0.00	0.00	0.00	0.00	0.00	0.00	0.00
**BP86**	IS	-18.32	-17.83	24.79	22.45	23.40	22.21	24.44	24.25	-3.54	-3.77	1.07	-1.24
	HS	-25.52	-26.90	54.60	48.27	30.11	33.90	39.26	36.11	16.22	15.61	13.75	7.66
	LS	0.00	0.00	0.00	0.00	0.00	0.00	0.00	0.00	0.00	0.00	0.00	0.00
**OPBE**	IS	-27.30	-27.92	24.76	23.93	19.73	20.34	21.01	20.39	-11.39	-13.06	-2.65	-6.00
	HS	-40.30	-42.48	55.18	49.16	20.08	20.33	28.18	23.68	0.92	-1.07	7.09	-0.60
	LS	0.00	0.00	0.00	0.00	0.00	0.00	0.00	0.00	0.00	0.00	0.00	0.00
**TPSS**	IS	-18.04	-17.77	21.88	19.31	17.84	15.73	18.39	15.63	-3.80	-3.44	-1.34	-3.97
	HS	-27.15	-28.66	49.29	42.29	28.73	26.01	36.96	33.78	14.13	14.25	10.61	4.52
	LS	0.00	0.00	0.00	0.00	0.00	0.00	0.00	0.00	0.00	0.00	0.00	0.00
**M06-L**	IS	-30.73	-32.08	18.16	14.88	12.69	11.26	13.28	19.19	-7.28	-4.86	-9.21	-12.71
	HS	-53.58	-56.29	31.73	23.35	2.29	0.40	5.50	2.07	-6.99	-3.70	-7.46	-17.43
	LS	0.00	0.00	0.00	0.00	0.00	0.00	0.00	0.00	0.00	0.00	0.00	0.00
**r^2-SCAN**	IS	-28.45	-30.82	16.26	13.68	16.46	44.94	19.85	21.43	-11.61	-12.15	0.59	-0.78
	HS	-24.73	-42.34	40.42	35.37	62.15	58.41	36.46	16.79	12.44	9.00	3.64	1.84
	LS	0.00	0.00	0.00	0.00	0.00	0.00	0.00	0.00	0.00	0.00	0.00	0.00
**B3LYP**	IS	-30.26	-28.89	13.64	10.56	7.78	8.36	14.23	15.83	-12.46	-11.89	-9.72	-9.77
	HS	-45.08	-45.42	26.67	18.40	5.38	6.11	9.26	5.90	-4.77	-3.95	-1.47	-9.02
	LS	0.00	0.00	0.00	0.00	0.00	0.00	0.00	0.00	0.00	0.00	0.00	0.00
**B3LYP***	IS	-29.60	-25.97	13.84	13.52	8.61	20.08	13.75	16.81	-12.96	-10.27	-15.71	-15.02
	HS	-44.03	-40.82	27.13	24.70	6.36	9.67	8.90	11.37	-3.55	1.79	-4.76	-5.86
	LS	0.00	0.00	0.00	0.00	0.00	0.00	0.00	0.00	0.00	0.00	0.00	0.00
**PBE0**	IS	-38.54	-37.71	10.36	7.59	17.04	5.31	3.40	23.27	-18.70	-19.36	-14.06	-12.50
	HS	-55.18	-55.37	22.58	15.23	-2.86	-2.50	1.78	2.47	-15.75	-15.58	-10.35	-14.33
	LS	0.00	0.00	0.00	0.00	0.00	0.00	0.00	0.00	0.00	0.00	0.00	0.00
**TPSSh**	IS	-24.71	-24.16	17.02	14.45	21.28	14.23	16.69	13.45	-8.35	-11.07	-7.70	-10.98
	HS	-37.12	-38.17	38.76	31.66	16.10	16.53	25.40	22.69	4.12	1.44	2.46	-3.71
	LS	0.00	0.00	0.00	0.00	0.00	0.00	0.00	0.00	0.00	0.00	0.00	0.00
**M05**	IS	-52.05	-51.30	12.45	9.80	-1.27	-0.07	21.27	-7.44	-22.98	-22.53	-17.92	-23.45
	HS	-46.72	-73.36	17.53	9.57	-19.55	-19.01	-18.06	-24.70	-27.20	-25.07	-28.45	-35.03
	LS	0.00	0.00	0.00	0.00	0.00	0.00	0.00	0.00	0.00	0.00	0.00	0.00
**M06**	IS	-40.64	-39.58	12.69	8.65	22.25	2.09	7.82	13.23	-20.97	-19.15	-18.07	-5.76
	HS	-65.79	-64.90	17.78	9.54	-14.08	-13.99	-8.19	-12.42	-20.66	-17.40	-24.03	13.34
	LS	0.00	0.00	0.00	0.00	0.00	0.00	0.00	0.00	0.00	0.00	0.00	0.00
**ω-B97XD**	IS	-33.62	6.52	17.38	11.42	23.48	7.58	11.23	27.53	-12.98	-4.45	-16.07	0.30
	HS	-25.49	-43.16	27.25	20.50	29.04	3.56	8.86	23.94	17.49	18.23	-12.86	-18.01

NS – No solvente; WS – with Solvent

Continuation of Table S8.

Complex		7		8		9		10		11		12	
Exp. Spin (S)		0		0		2		2		1		3/2	
		NS	WS	NS	WS	NS	WS	NS	WS	NS	WS	NS	WS
BP86	LS	0.00	0.00	0.00	0.00	0.00	0.00	0.00	0.00	0.00	0.00	0.00	0.00
	IS	16.02	14.01	17.61	14.10	-11.76	-12.63	-29.16	-28.35	-8.53	-7.35	5.57	5.60
	HS	33.82	27.01	40.11	33.78	-15.74	-39.30	-25.54	-26.02	16.63	17.23	24.25	21.35
OPBE	LS	0.00	0.00	0.00	0.00	0.00	0.00	0.00	0.00	0.00	0.00	0.00	0.00
	IS	16.19	14.22	19.62	16.77	-15.67	-16.61	-33.13	-32.87	-8.74	-7.53	0.72	0.14
	HS	33.90	26.96	40.97	34.96	-24.05	-27.20	-21.41	-22.77	15.62	13.33	14.79	11.53
TPSS	LS	0.00	0.00	0.00	0.00	0.00	0.00	0.00	0.00	0.00	0.00	0.00	0.00
	IS	17.18	14.93	18.41	15.23	-15.05	-13.81	-32.49	-31.71	-9.57	-8.81	1.61	2.31
	HS	30.85	23.93	37.35	31.40	-25.65	-21.99	-30.84	-31.54	12.35	11.04	19.35	17.01
M06-L	LS	0.00	0.00	0.00	0.00	0.00	0.00	0.00	0.00	0.00	0.00	0.00	0.00
	IS	11.59	9.62	14.32	10.75	-13.25	-14.77	-22.72	-26.37	-16.34	-15.45	-10.46	-12.70
	HS	15.58	9.37	20.68	13.36	-30.92	-33.03	-37.80	-43.82	-10.77	-12.86	-0.10	-3.85
r^2-SCAN	LS	0.00	0.00	0.00	0.00	0.00	0.00	0.00	0.00	0.00	0.00	0.00	0.00
	IS	10.44	7.07	13.10	10.58	-12.65	-13.76	-23.30	-24.04	-12.54	-13.69	-5.30	-7.00
	HS	26.26	20.62	28.91	25.02	-29.90	-31.46	-32.85	-34.02	28.05	20.42	8.32	5.26
B3LYP	LS	0.00	0.00	0.00	0.00	0.00	0.00	0.00	0.00	0.00	0.00	0.00	0.00
	IS	5.42	3.54	11.72	8.50	-16.94	-16.52	-39.10	-39.28	-15.84	-15.06	-9.32	-24.95
	HS	12.11	6.00	14.44	8.40	-31.20	-28.81	-50.30	-53.71	-4.59	-8.72	-2.68	-15.35
B3LYP*	LS	0.00	0.00	0.00	0.00	0.00	0.00	0.00	0.00	0.00	0.00	0.00	0.00
	IS	5.72	6.67	2.46	2.65	-17.79	-16.33	-38.70	-36.61	-15.25	-12.96	-9.84	-10.29
	HS	4.79	2.96	14.48	13.28	-34.35	-33.47	-50.11	-48.36	-3.88	-2.32	-3.03	-1.70
PBE0	LS	0.00	0.00	0.00	0.00	0.00	0.00	0.00	0.00	0.00	0.00	0.00	0.00
	IS	4.66	2.13	9.07	6.22	-21.96	-21.51	-45.18	-44.65	-18.03	-17.41	-14.07	-16.41
	HS	8.60	2.65	11.87	6.56	-43.01	-45.36	-63.25	-66.05	-13.93	-13.93	-3.73	-25.72
TPSSh	LS	0.00	0.00	0.00	0.00	0.00	0.00	0.00	0.00	0.00	0.00	0.00	0.00
	IS	13.33	11.43	14.54	11.01	-17.90	-17.57	-38.74	-37.83	-12.68	-11.69	-4.67	-6.66
	HS	21.31	13.93	27.68	21.90	-26.91	-24.08	-43.34	-44.15	6.51	2.65	6.33	2.58
M05	LS	0.00	0.00	0.00	0.00	0.00	0.00	0.00	0.00	0.00	0.00	0.00	0.00
	IS	-0.53	-4.25	7.71	4.64	-21.38	-21.65	-37.80	-39.17	-24.29	-25.90	-18.93	-19.34
	HS	-0.76	-7.87	-1.69	-8.30	-51.60	-55.92	-59.16	-62.52	-18.97	-28.18	-13.37	-13.62
M06	LS	0.00	0.00	0.00	0.00	0.00	0.00	0.00	0.00	0.00	0.00	0.00	0.00
	IS	1.96	-0.89	14.09	8.88	-23.75	-22.09	-40.16	-38.66	-18.92	-19.40	-13.82	-16.26
	HS	4.92	-1.25	9.93	3.28	-48.18	-50.07	-59.85	-61.61	-12.21	-20.29	-10.73	-9.47
ω-B97XD	LS	0.00	0.00	0.00	0.00	0.00	0.00	0.00	0.00	0.00	0.00	0.00	0.00
	IS	4.32	2.07	9.83	5.91	-17.97	-17.52	-32.97	-32.42	-17.15	-15.79	-9.40	-32.90
	HS	58.52	52.62	41.50	25.17	-36.92	-39.84	-49.20	-53.34	-12.66	-11.21	-2.36	-26.72

NS – No solvente; WS – with Solvent
